# Supplementary material for: Mitochondrial protein C15ORF48 is a stress-independent inducer of autophagy that regulates oxidative stress and autoimmunity
Source: Nat Commun. 2024 Feb 1;15:953. doi: 10.1038/s41467-024-45206-1 (PMC10831050; doi:10.1038/s41467-024-45206-1)
Supplement: Supplementary file 1 — Supplementary Information [file 41467_2024_45206_MOESM1_ESM.pdf]

Supplementary Information

Mitochondrial protein C15ORF48 is a stress-independent inducer of autophagy that regulates oxidative stress and autoimmunity

Yuki Takakura[1,2,3], Moeka Machida[1,2], Natsumi Terada[1,2], Yuka Katsumi[1], Seika Kawamura[1], Kenta Horie[3], Maki Miyauchi[3,4], Tatsuya Ishikawa[3,4], Nobuko Akiyama[3], Takao Seki[3], Takahisa Miyao[3,4], Mio Hayama[3,4], Rin Endo[3,4], Hiroto Ishii[3,4], Yuya Maruyama[3,4], Naho Hagiwara[3], Tetsuya J. Kobayashi[5], Naoto Yamaguchi[2], Hiroyuki Takano[1], Taishin Akiyama[3,4]*, and Noritaka Yamaguchi[1,2,3]*

[1]Department of Molecular Cardiovascular Pharmacology, Graduate School of Pharmaceutical Sciences, Chiba University, Chiba 260-8675, Japan.

[2]Laboratory of Molecular Cell Biology, Graduate School of Pharmaceutical Sciences, Chiba University, Chiba 260-8675, Japan.

[3]Laboratory for Immune Homeostasis, RIKEN Center for Integrative Medical Sciences, Yokohama 230-0045, Japan.

[4]Immunobiology, Graduate School of Medical Life Science, Yokohama City University, Yokohama 230-0045, Japan.

[5]Institute of Industrial Science, The University of Tokyo, Tokyo 153-8505, Japan

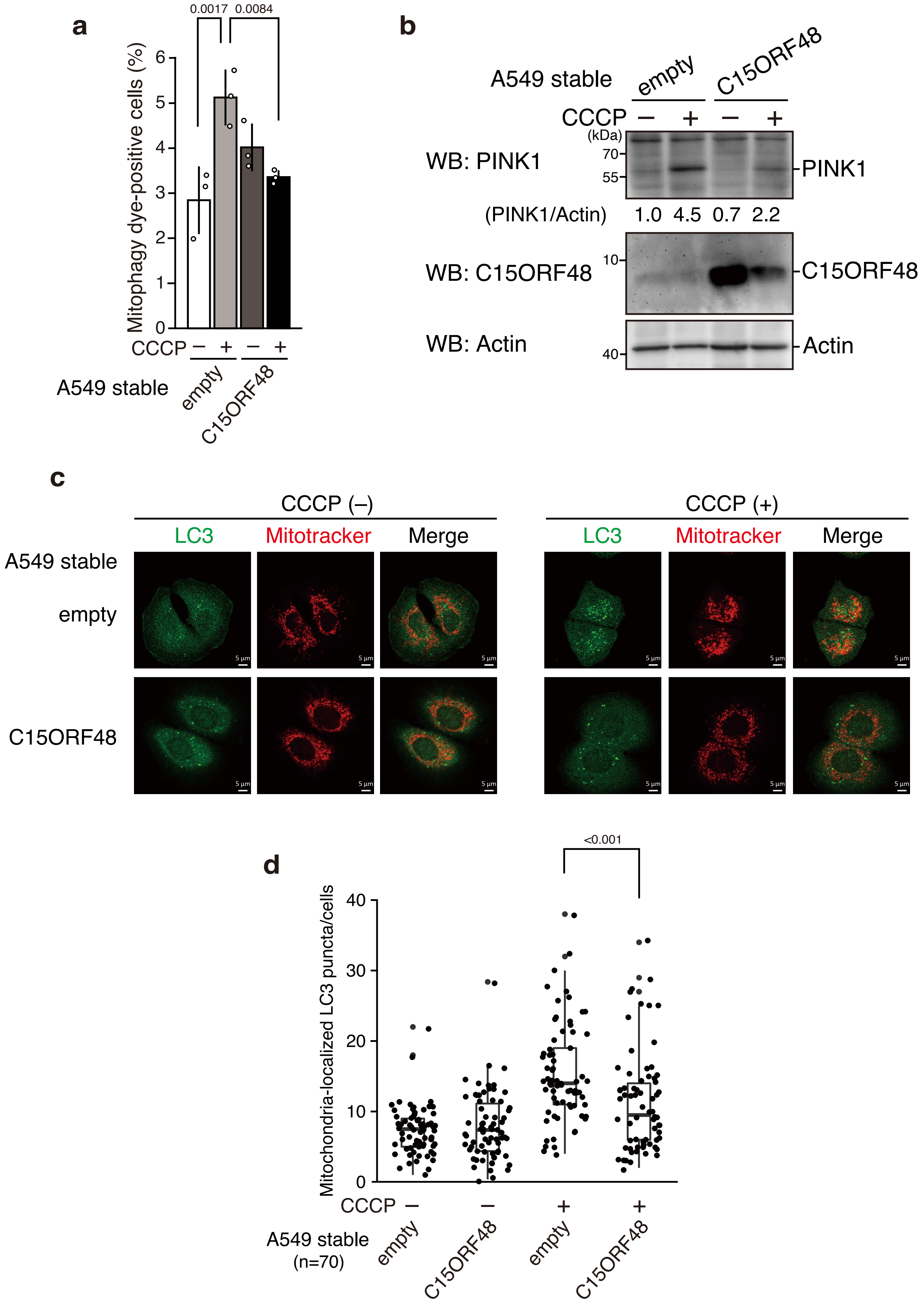

Supplementary Fig.1

Supplementary Fig. 1: C15ORF48 has a suppressive role in mitophagy.

a, A549/empty and A549/C15ORF48 cells were treated or left untreated with CCCP (50 μM) for 6 h after replacement of culture media and then stained with Mitophagy dye for 30 min. After incubation, Mitophagy dye-positive cells were detected by flow cytometry. Statistical significance was calculated using two-way ANOVA followed by Tukey's multiple comparisons test (n = 3, biological replicates).

b, A549/empty and A549/C15ORF48 cells were treated or left untreated with CCCP (50 μM) for 6 h after replacement of culture media and then subjected to western blotting with the indicated antibodies. Band intensities were measured, and quantitative ratios are shown. Data are representative of three independent experiments with three biological replicates.

c,**d**, A549/empty and A549/C15ORF48 cells were treated or left untreated with CCCP (50 μM) for 6 h after replacement of culture media. Cells were fixed and stained with anti-LC3 antibodies and MitoTracker (**c**). Numbers of LC3 puncta colocalized with mitochondria in each cell were calculated and are shown as means ± SDs. Statistical significance was calculated using two-way ANOVA followed by Tukey's multiple comparisons test (n = 70 cells from two independent experiments) (**d**)

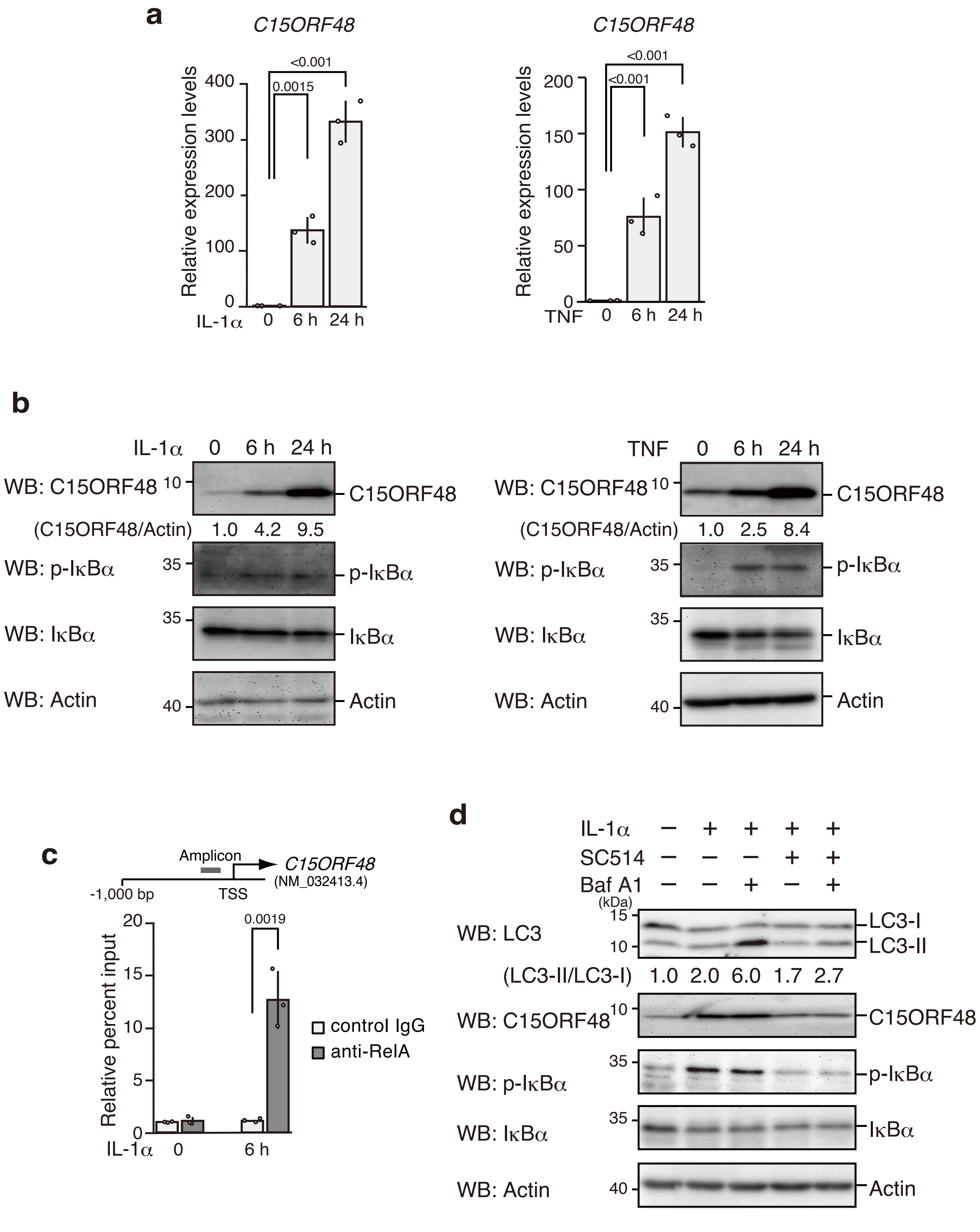

Supplementary Fig.2

Supplementary Fig. 2: Induction of *C15ORF48* expression by NF-κB signaling.

a, A549 cells were stimulated with IL-1α (10 ng/mL) or TNF (10 ng/mL) for the indicated times. After incubation, cells were analyzed for *C15ORF48* mRNA expression by qPCR. *GAPDH* mRNA expression was used to normalize these data. The expression level of *C15ORF48* mRNA in unstimulated cells was set to 1. Results represent the mean ± SD. Statistical significance was calculated using two-way ANOVA followed by Tukey's multiple comparisons test (n = 3, biological replicates).

b, A549 cells were stimulated with IL-1α (10 ng/mL) or TNF (10 ng/mL) for the indicated times. After incubation, cells were subjected to western blotting with the indicated antibodies. Data are representative of three independent experiments with three biological replicates.

c, Schematic diagram of *C15ORF48* promoter region (upper). The region targeted by the designed primer pair is shown (Amplicon). TSS, transcription start site. A549 cells were stimulated with IL-1α (10 ng/mL) for the indicated times. After incubation, cells were analyzed by ChIP assays with anti-RelA or control antibodies to evaluate the binding activity of RelA to the *C15ORF48* promoter region (lower). Percentage input values are shown relative to those of control immunoprecipitates. These results represent the mean ± SD. Statistical significance was calculated using two-tailed unpaired Student's *t*-test (n = 3, biological replicates).

d, A549 cells were stimulated with IL-1α (10 ng/mL) or left untreated for 24 h. 21 h after stimulation, cells were treated with the NF-κB inhibitor SC514 (100 μM) or without. Some samples were further treated with bafilomycin A1 (Baf A1, 200 μM) for the final 1 h. After incubation, cells were subjected to western blotting with the indicated antibodies. Data are representative of three independent experiments with three biological replicates.

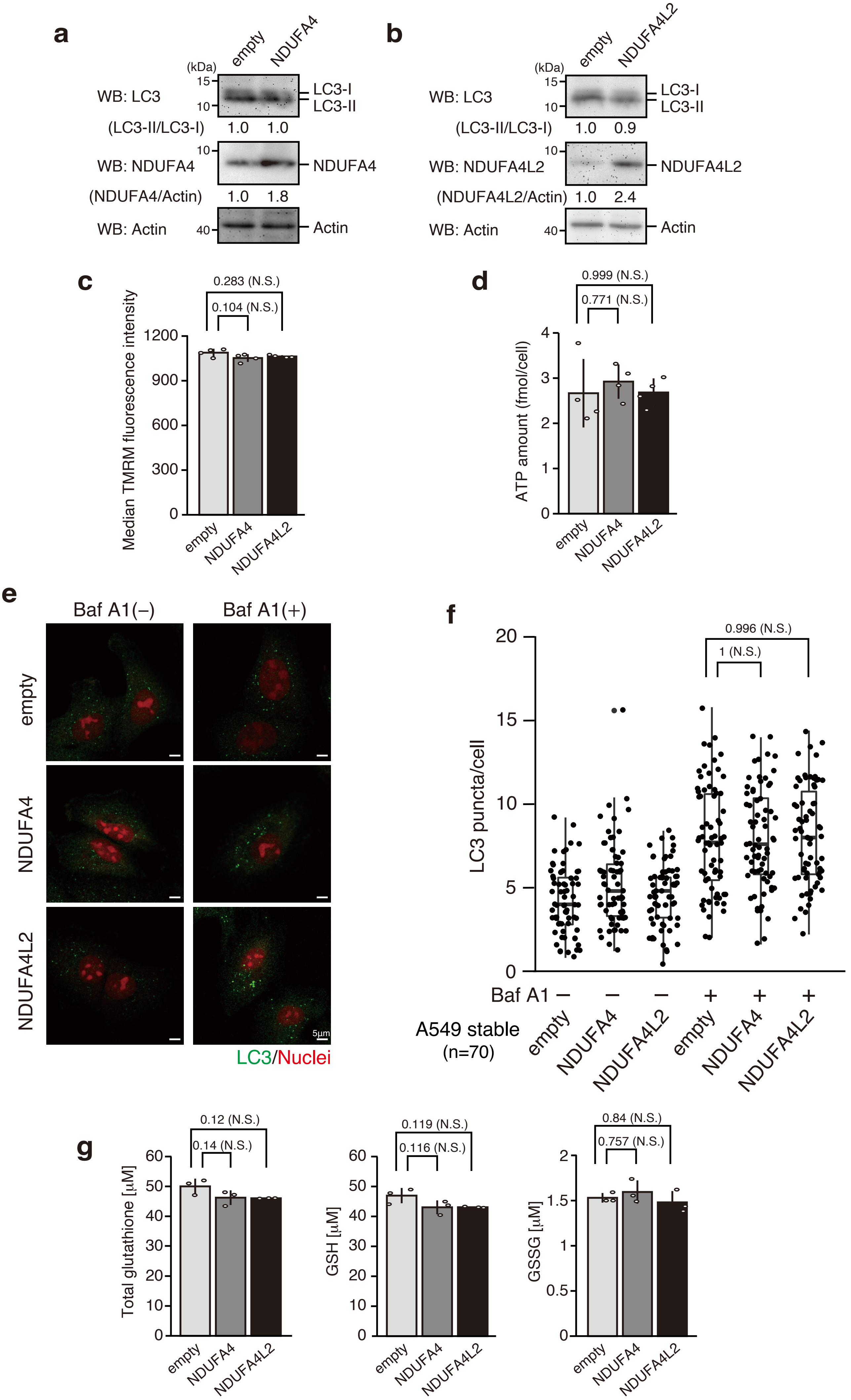

Supplementary Fig.3

Supplementary Fig. 3: Forced expression of NDUFA4 or NDUFA4L2 did not induce autophagy.

a,**b**, A549 cells stably expressing NDUFA4 (A549/NDUFA4) or NDUFA4L2 (A549/NDUFA4L2) and A549/empty were lysed and subjected to western blotting with the indicated antibodies. Data are representative of three independent experiments with three biological replicates.

c, A549/empty, A549/NDUFA4, and A549/NDUFA4L2 cells were analyzed for mitochondrial membrane potential by staining with TMRM (30 nM) for 30 min. Median TMRM fluorescence intensities are shown as the mean ± SD. Statistical significance was calculated using two-way ANOVA followed by Tukey's multiple comparisons test (n = 4, biological replicates). Not significant, N.S.

d, A549/empty, A549/NDUFA4, and A549/NDUFA4L2 cells were analyzed for intracellular ATP levels. ATP concentrations are shown as the mean ± SD. Statistical significance was calculated using two-way ANOVA followed by Tukey's multiple comparisons test (n = 4, biological replicates). Not significant, N.S.

e,**f**, A549/empty, A549/NDUFA4, and A549/NDUFA4L2 cells were treated with bafilomycin A1 (Baf A1, 200 μM, 1 h) or left untreated 3 h after replacement of culture media. Cells were fixed and stained with anti-LC3 antibodies. Nuclei were counter-stained with PI (**e**). Numbers of LC3 puncta in each cell were calculated and are shown as means ± SDs. Statistical significance was calculated using two-way ANOVA followed by Tukey's multiple comparisons test (n = 70 cells from two independent experiments). Not significant, N.S. (**f**).

g, A549/empty, A549/NDUFA4, and A549/NDUFA4L2 cells were subjected to glutathione assays. Glutathione concentrations were determined and are shown as means ± SDs. Statistical significance was calculated using two-way ANOVA followed by Tukey's multiple comparisons test (n = 3, biological replicates). Not significant, N.S.

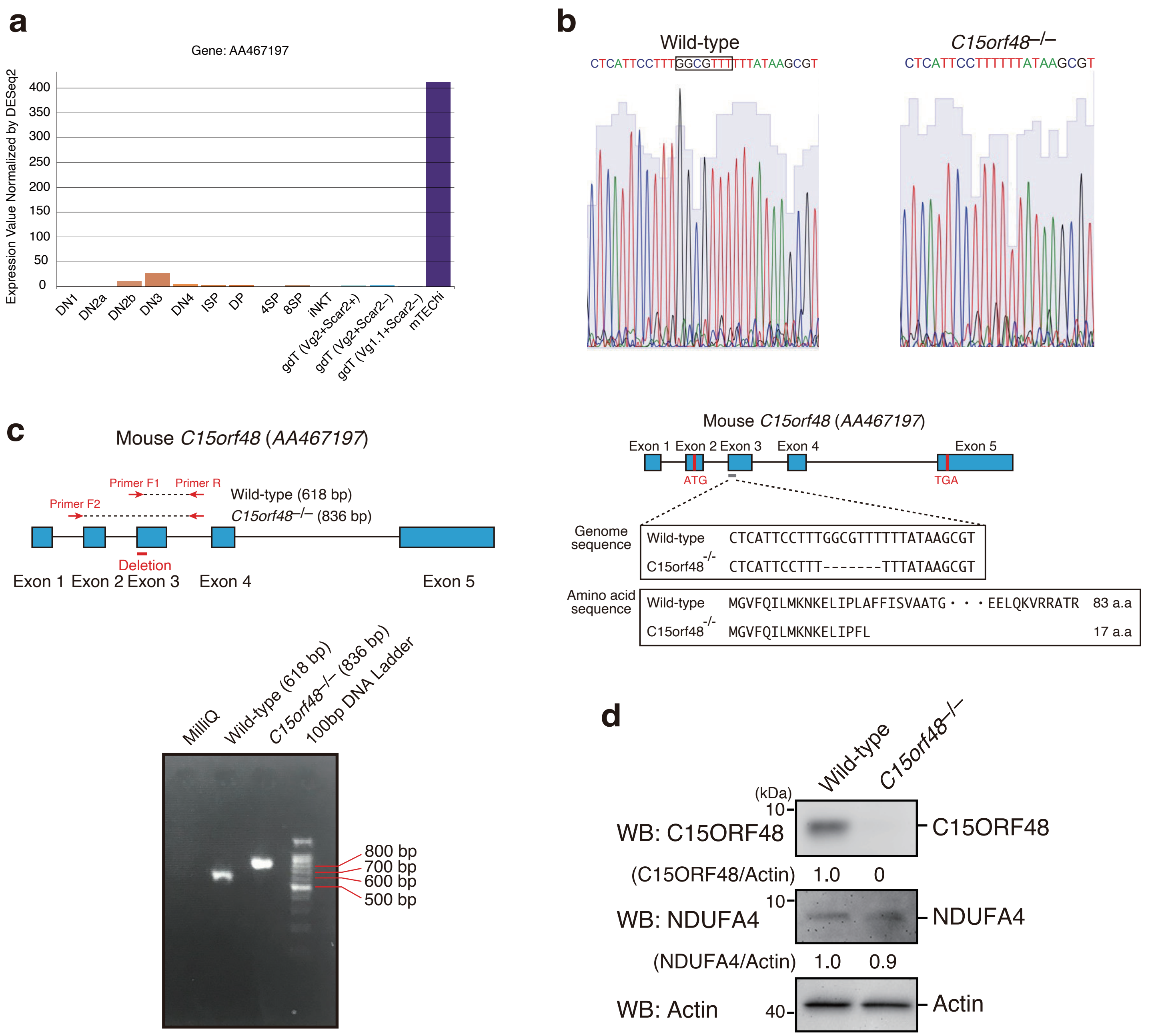

Supplementary Fig.4

Supplementary Fig. 4: A 7-bp deletion in the exon 3 of *C15orf48* gene in *C15orf48*$^{-/-}$ mice.

a, Expression levels of mouse *C15orf48* (*AA467197*) in thymic cells in immunological genome project platform data.

b, Sequencing data of CRISPR-Cas9-targeted regions in *C15orf48* exon 3 from wild-type and *C15orf48*$^{-/-}$ mice. The boxed sequence indicates a 7-bp deletion in *C15orf48*$^{-/-}$ mice (upper). The deleted region in *C15orf48*$^{-/-}$ mice is shown as a gray bar. *C15orf48* genome sequences and corresponding amino acid sequences of wild-type and *C15orf48*$^{-/-}$ mice are shown (lower).

c, Schematic diagram of *C15orf48* genomic structure. Primer F1 recognizes the region corresponding to the 7-bp deletion in *C15orf48*$^{-/-}$ mice; therefore, the primer pair between F1 and R cannot amplify PCR products from *C15orf48*$^{-/-}$ mouse genomic DNA (upper). A representative image of genotyping PCR of wild-type and *C15orf48*$^{-/-}$ mice using three primers (Primer F1, F2, and R) (lower).

d, Extracts from testes of wild-type and *C15orf48*$^{-/-}$ mice were subjected to western blotting with the indicated antibodies. Data are representative of three independent experiments with three biological replicates.

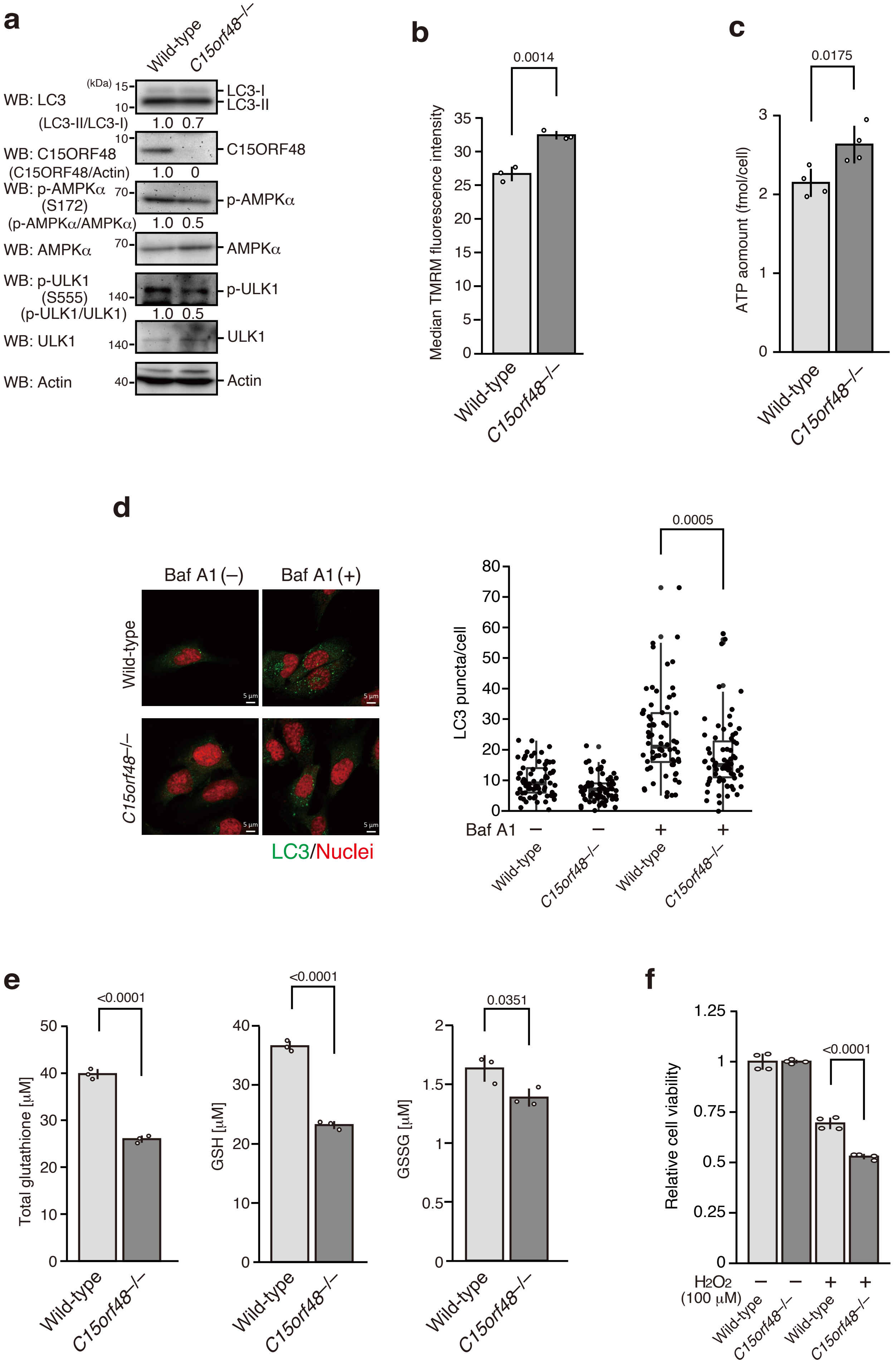

Supplementary Fig.5

Supplementary Fig. 5: Primary fibroblasts from *C15orf48*$^{-/-}$ mice show reduction in basal autophagy.

a, Primary fibroblasts from littermate wild-type and *C15orf48*$^{-/-}$ embryos were lysed 3 h after replacement of culture media and subjected to western blotting with the indicated antibodies.

b, Primary fibroblasts from littermate wild-type and *C15orf48*$^{-/-}$ embryos were analyzed for mitochondrial membrane potential by staining with TMRM (30 nM) for 30 min. Median TMRM fluorescence intensities are shown as the mean ± SD. Statistical significance was calculated using two-tailed unpaired Student's *t*-test (n = 3, biological replicates).

c, Primary fibroblasts from littermate wild-type and *C15orf48*$^{-/-}$ embryos were analyzed for intracellular ATP levels. ATP amount in a cell is shown as the mean ± SD. Statistical significance was calculated using two-tailed unpaired Student's *t*-test (n = 4, biological replicates).

d, Primary fibroblasts from littermate wild-type and *C15orf48*$^{-/-}$ embryos were treated with bafilomycin A1 (Baf A1, 200 μM, 1 h) or left untreated 3 h after replacement of culture media. Cells were fixed and stained with anti-LC3 antibodies. Nuclei were counter-stained with PI (left). Numbers of LC3 puncta in each cell were calculated and are shown as means ± SDs. Statistical significance was calculated using two-way ANOVA followed by Tukey’s multiple comparisons test (n = 70 cells from two independent experiments) (right).

e, Primary fibroblasts from littermate wild-type and *C15orf48*$^{-/-}$ embryos were subjected to glutathione assays. Glutathione concentrations were determined and are shown as means ± SDs. Statistical significance was calculated using two-tailed unpaired Student's *t*-test (n = 3, biological replicates).

f, Primary fibroblasts from littermate wild-type and *C15orf48*$^{-/-}$ embryos were seeded at 20,000 cells/well and treated with or without H_2O_2 (100 μM) for 18 h. After incubation, cell viability was calculated with an MTT assay. The quantitative ratio of MTT absorbance in H_2O_2-treated cells normalized to that in untreated cells was calculated and is shown as the mean ± SD. Statistical significance was calculated using two-tailed unpaired Student's *t*-test (n = 4, biological replicates).

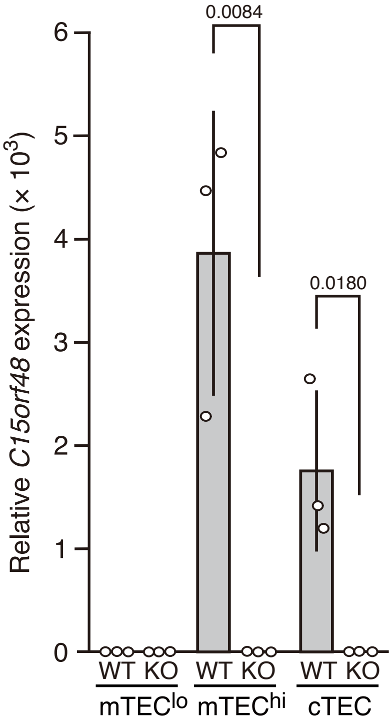

Supplementary Fig.6

Supplementary Fig. 6: Expression of *C15orf48* mRNA in mTEC^{hi} and cTEC.

mTEC^{lo}, mTEC^{hi}, and cTEC from wild-type and *C15orf48*$^{-/-}$ mice were analyzed for *C15orf48* mRNA expression by qPCR. *36B4* mRNA expression was used to normalize these data. The expression level of *C15orf48* mRNA in wild-type mTEC^{lo} was set to 1. Results represent the mean ± SD. Statistical significance as calculated using two-tailed unpaired Student's *t*-test (n = 3, biological replicates). Not significant, N.S.

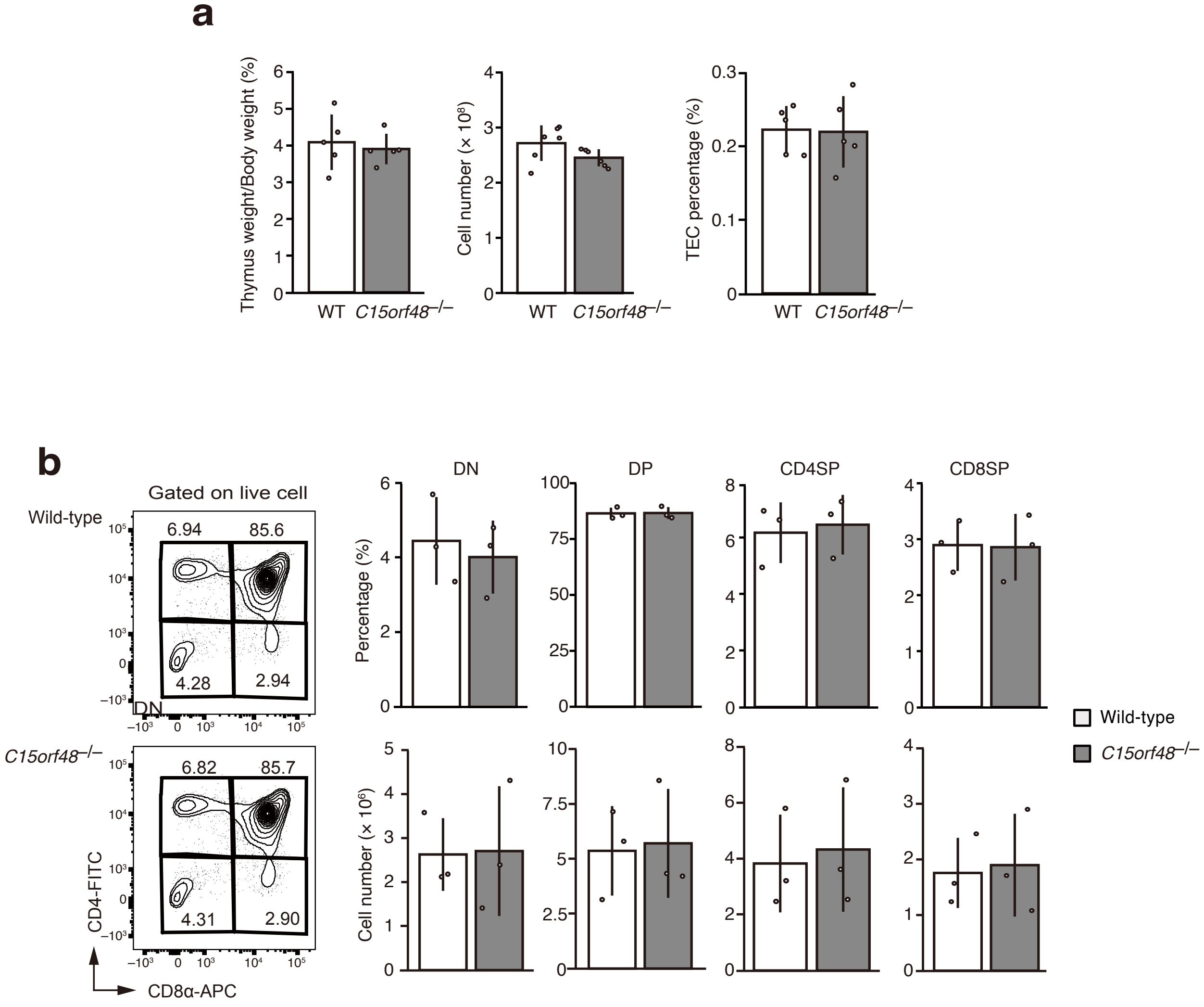

Supplementary Fig.7

Supplementary Fig. 7: Development of thymocytes in wild-type and *C15orf48*$^{-/-}$ mice.

a, Ratios of thymus weight to whole body weight (left), total cell numbers in thymus (middle), and ratios of TECs to whole thymic cells (right) in 4-week-old wild-type and *C15orf48*$^{-/-}$ mice are shown as means ± SDs. Statistical significance was calculated using Student's *t*-test (n = 5, biological replicates).

b, Representative images of flow cytometry plots of DN, DP, CD4SP, and CD8SP thymocytes in 4-week-old wild-type and *C15orf48*$^{-/-}$ mice (left). Numbers of DN, DP, CD4SP, and CD8SP cells and their ratios to total thymocytes are shown as means ± SDs. Statistical significance was calculated using Student’s *t*-test (n = 3, biological replicates) (right).

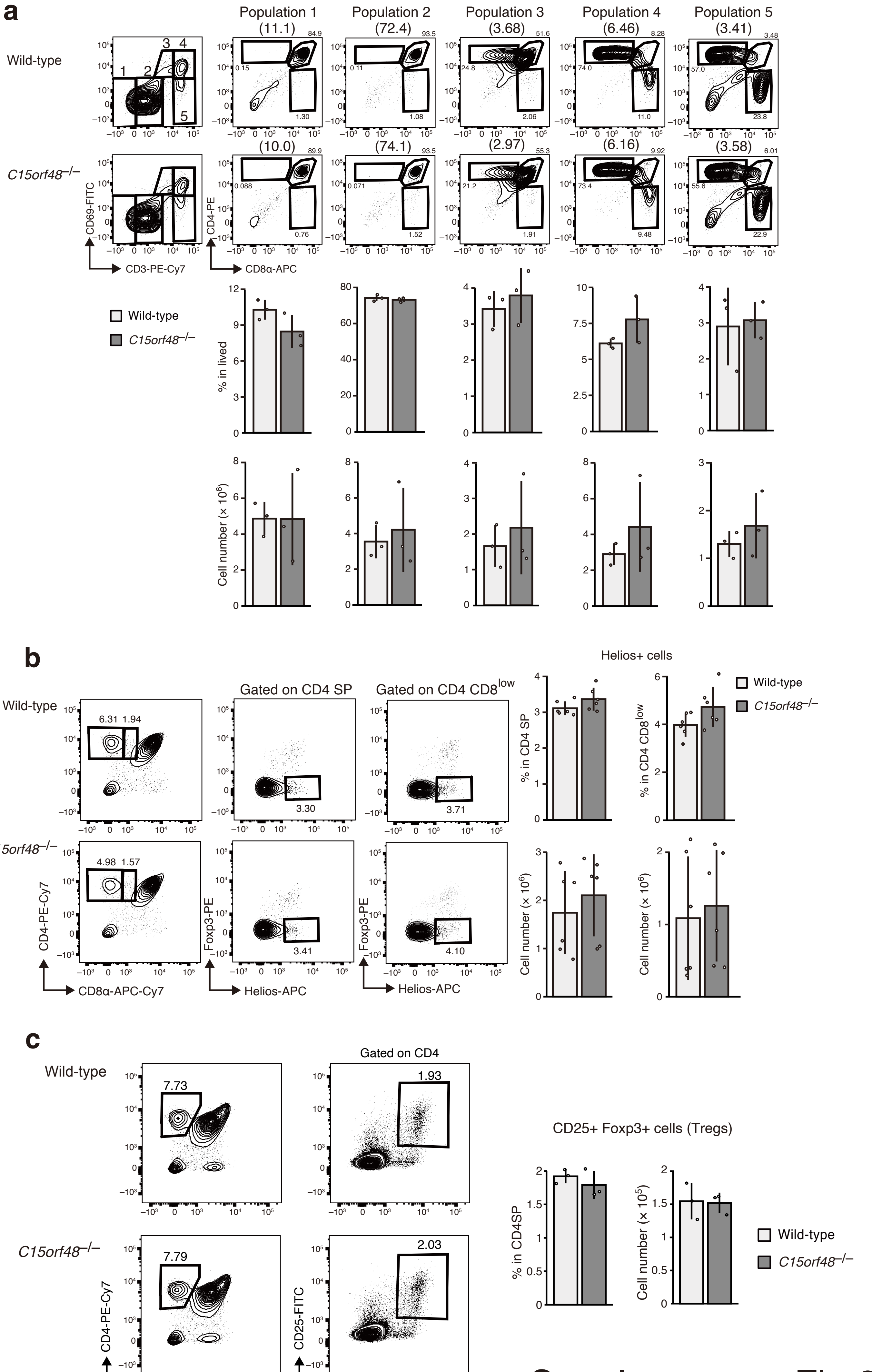

Supplementary Fig.8

Supplementary Fig. 8: Positive, negative, and Treg selections in wild-type and *C15orf48*$^{-/-}$ mice.

a, Surface staining of CD3 and CD69 of total thymocytes from 4-week-old wild-type and *C15orf48*$^{-/-}$ mice (upper left). Numbers in outlined areas (upper left) indicate subpopulations gated at the upper right, and numbers in parentheses above the upper right-hand plots indicate percent thymocytes in each subpopulation. Numbers adjacent to outlined areas (upper right) indicate percent cells in each. Below, average number and percentage of each subpopulation. Data are presented as means ± SDs. Statistical significance was calculated using two-tailed unpaired Student's *t*-test (n = 3, biological replicates).

b, Representative images of flow cytometry plots of Helios-positive cells in CD4SP and CD4 CD8^{low} cells in 4-week-old wild-type and *C15orf48*$^{-/-}$ mice (left). Total numbers of Foxp3^{-} Helios^{+} cells and ratios of these cells to CD4SP or CD4 CD8^{low} cells are shown as means ± SDs. Statistical significance was calculated using two-tailed unpaired Student's *t*-test (n = 6, biological replicates).

c, Representative images of flow cytometry plots of CD25^{+} Foxp3^{+} cells in CD4SP cells in 4-week-old wild-type and *C15orf48*$^{-/-}$ mice (left). Total numbers of CD25^{+} Foxp3^{+} cells and ratios of these cells to CD4SP cells are shown as means ± SDs. Statistical significance was calculated using two-tailed unpaired Student's *t*-test (n = 3, biological replicates).

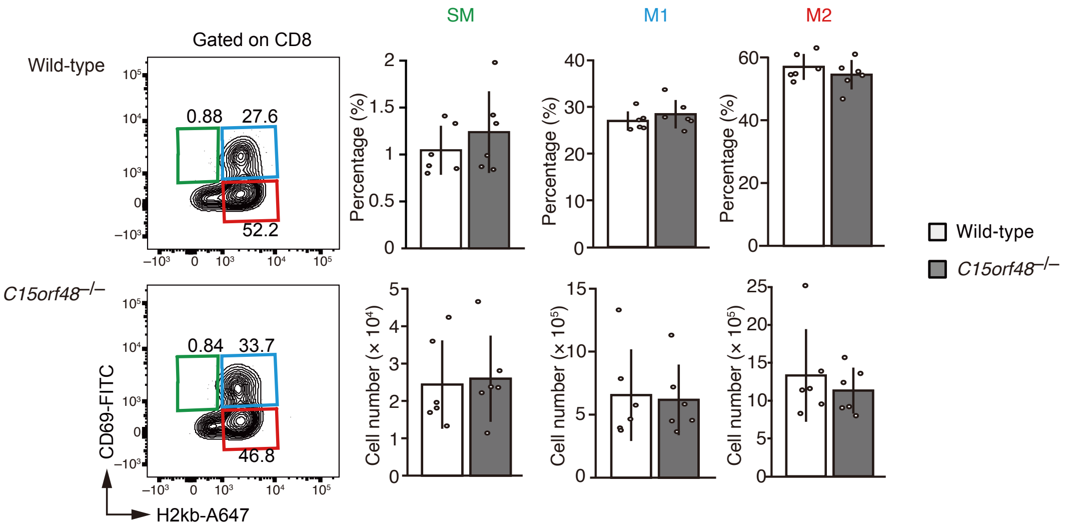

Supplementary Fig.9

Supplementary Fig. 9: SM, M1, and M2 CD8SP cells in wild-type and *C15orf48*$^{-/-}$ mice.

Representative images of flow cytometry plots of SM, M1, and M2 CD8SP cells in 4-week-old wild-type and *C15orf48*$^{-/-}$ mice (left). Numbers of SM, M1, and M2 CD8SP cells and their ratios to total thymocytes are shown as means ± SDs. Statistical significance was calculated using two-tailed unpaired Student's *t*-test (n = 7, biological replicates) (right).

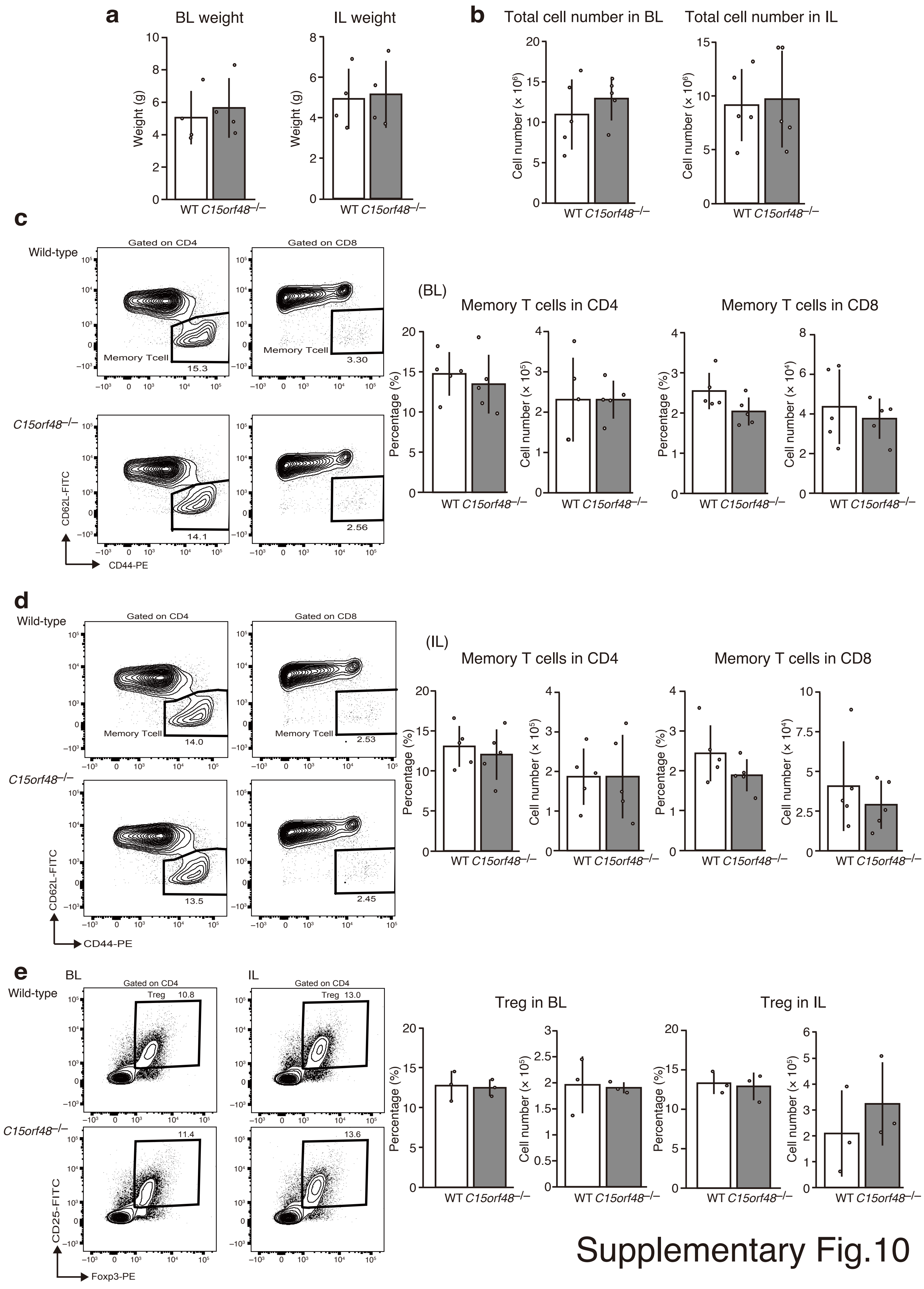

Supplementary Fig.10

Supplementary Fig. 10: Memory T cells and Tregs in secondary lymph nodes of wild-type and *C15orf48*$^{-/-}$ mice.

a,**b**, Weights (**a**) and total cell numbers (**b**) of brachial lymph nodes (BL) and inguinal lymph nodes (IL) and in 21-week-old wild-type and *C15orf48*$^{-/-}$ mice are shown as means ± SDs. Statistical significance was calculated using two-tailed unpaired Student's *t*-test (n = 3, biological replicates).

c, Representative images of flow cytometry plots of memory CD4 and CD8 T cells in BL in wild-type and *C15orf48*$^{-/-}$ mice. Total numbers of memory CD4 and CD8 T cells and ratios of these cells to total cell numbers in BL are shown as means ± SDs. Statistical significance was calculated using two-tailed unpaired Student's *t*-test (n = 3, biological replicates).

d, As in Extended Data Fig. 3c, except that memory CD4 and CD8 T cells in IL were analyzed.

e, As in Extended Data Fig. 3c, except that Tregs in BL and IL were analyzed.

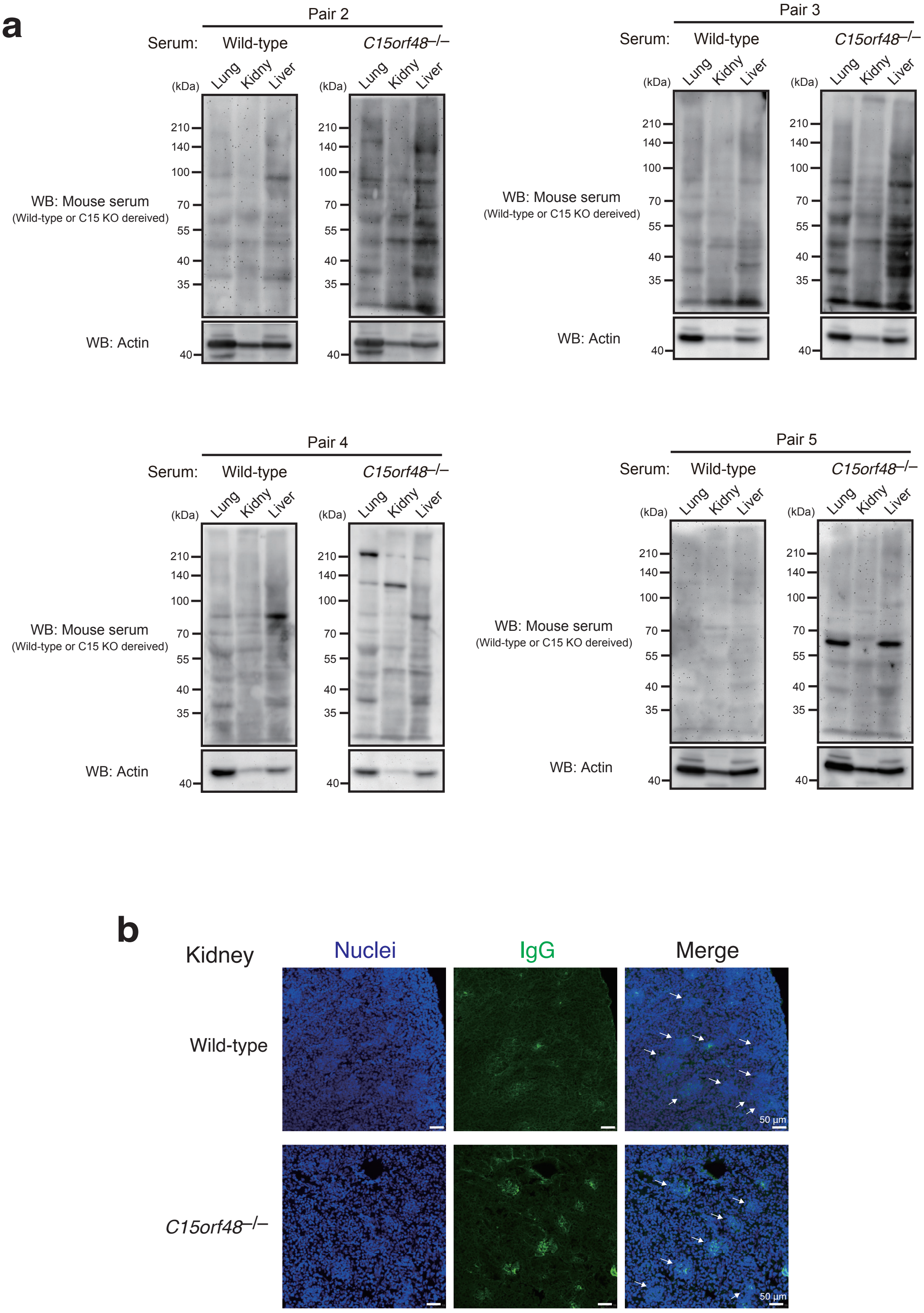

Supplementary Fig.11

Supplementary Fig. 11: Autoantibodies in sera from *C15orf48*$^{-/-}$ mice.

a, Western blotting of tissue lysates from *Rag1*$^{-/-}$ mice with sera from 21-week-old wild-type and *C15orf48*$^{-/-}$ littermate mice. All sera were diluted equally at 1:1000 with 5% skim-milk containing tris buffer. Western blotting using these sera was performed simultaneously to enable exact comparison of their immunoreactivities. Anti-actin blots were used as loading controls. Data show four independent experiments with sera from four different pairs of littermate mice.

b, Representative images of immunostaining of kidney sections from 21-week-old wild-type and *C15orf48*$^{-/-}$ mice with anti-mouse IgG at lower magnification. Nuclei were counter-stained with DAPI. Glomerulus-like cell clusters are shown by arrows.

a Related to Fig.1d

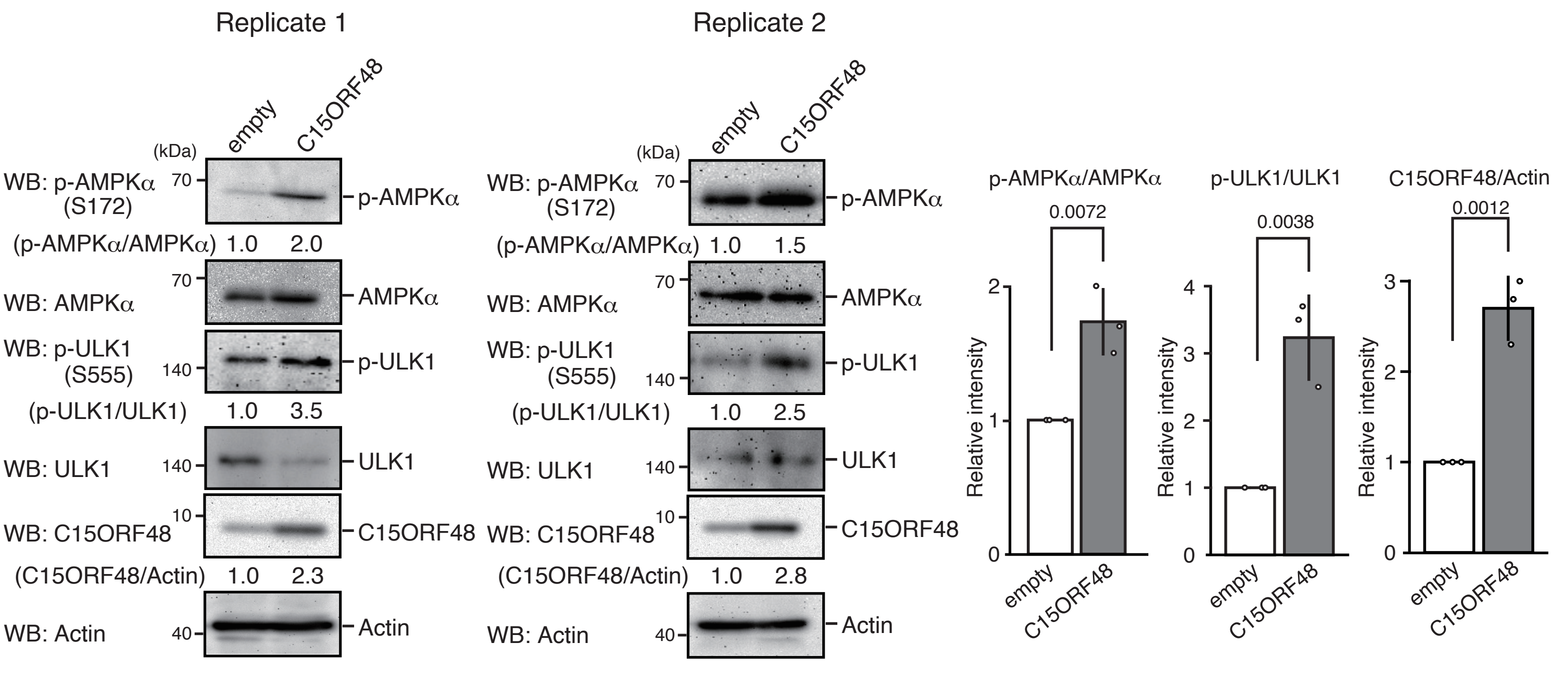

b Related to Fig.2a

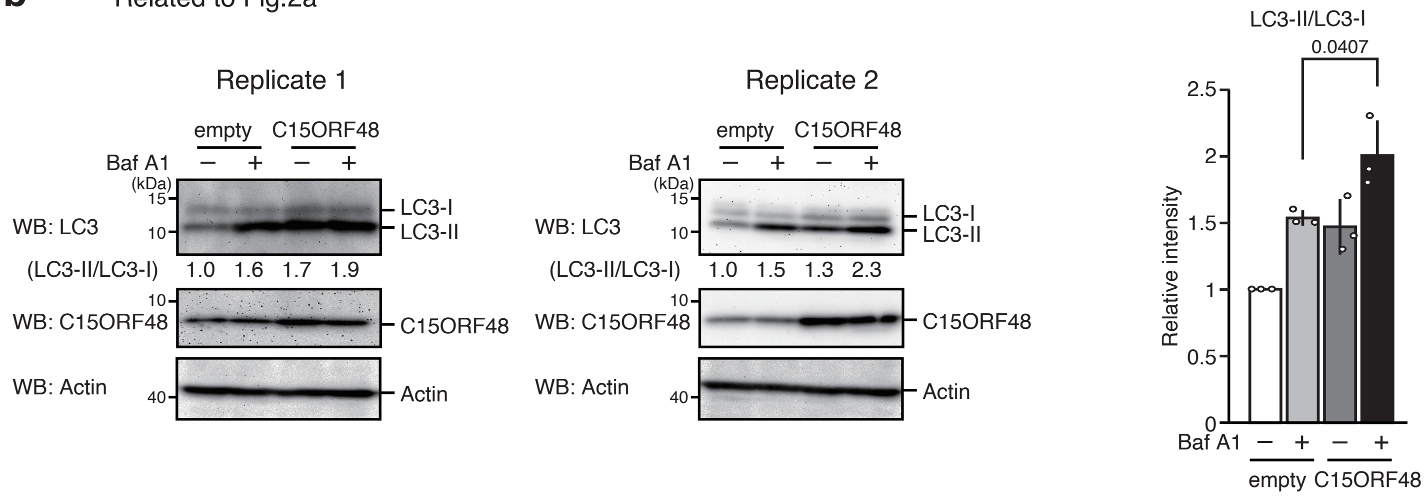

c Related to Fig.2d

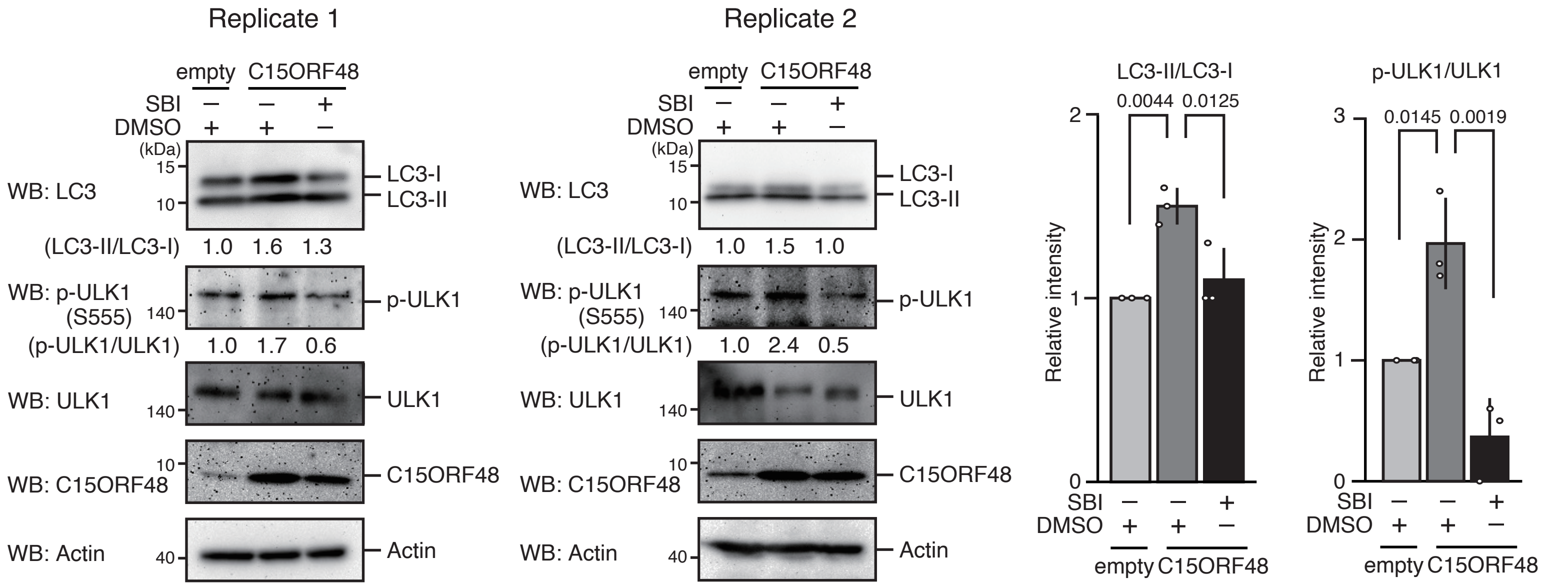

Supplementary Fig.12

Supplementary Fig. 12: Reproducibility and statistical analyses of western blotting.

a, Reproducibility and statistical analyses of Fig. 1d. Independent western blot data using independent samples are shown. Statistical significance was calculated using two-tailed unpaired Student's *t*-test (n = 3, biological replicates).

b, Reproducibility and statistical analyses of Fig. 2a. Independent western blot data using independent samples are shown. Statistical significance was calculated using two-way ANOVA followed by Tukey's multiple comparisons test (n = 3, biological replicates).

c, Reproducibility and statistical analyses of Fig. 2d. Independent western blot data using independent samples are shown. Statistical significance was calculated using two-way ANOVA followed by Tukey's multiple comparisons test (n = 3, biological replicates).

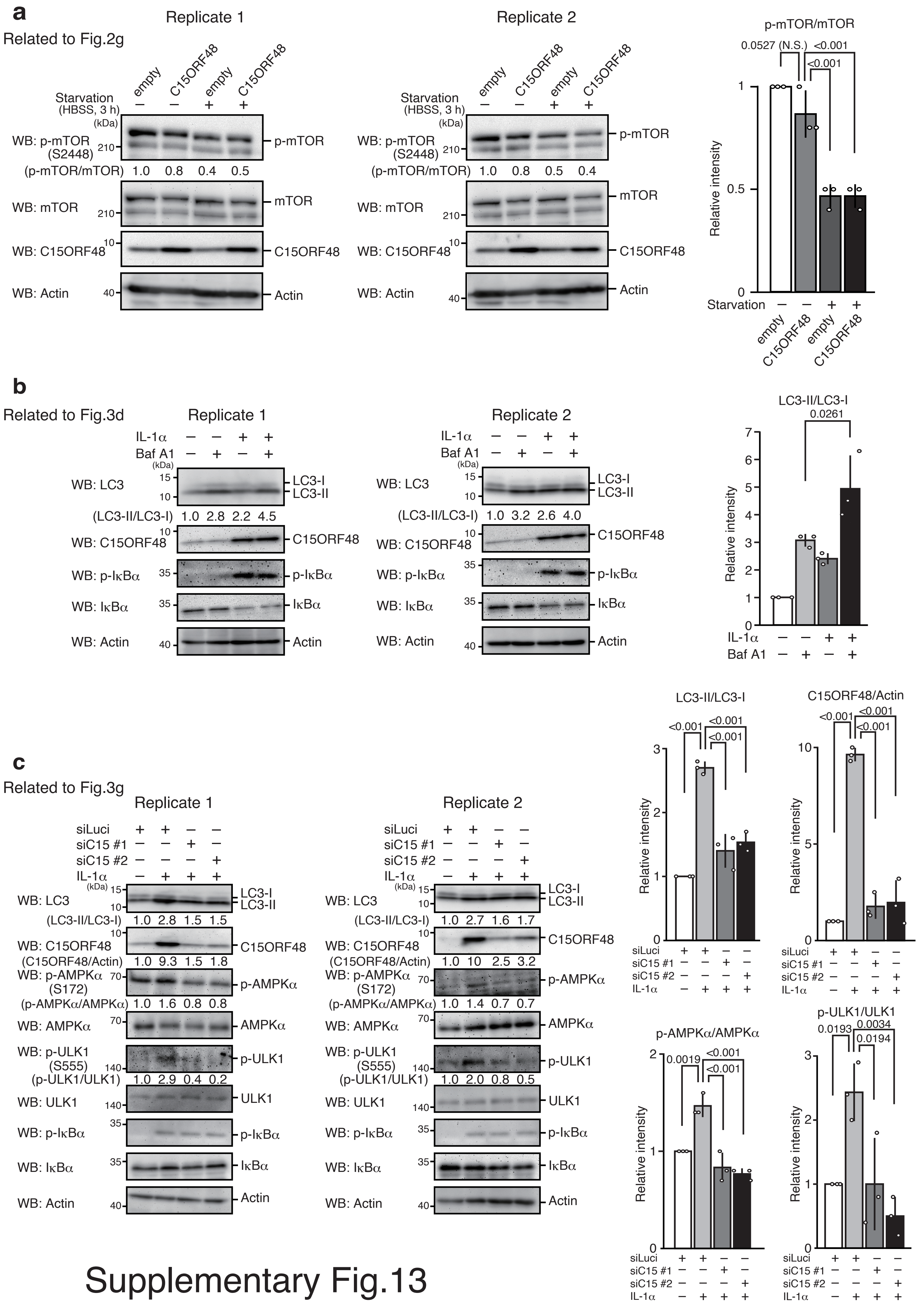

Supplementary Fig.13

Supplementary Fig. 13: Reproducibility and statistical analyses of western blotting.

a, Reproducibility and statistical analyses of Fig. 2g. Independent western blot data using independent samples are shown. Statistical significance was calculated using two-way ANOVA followed by Tukey's multiple comparisons test (n = 3, biological replicates). N.S., Not Significant.

b, Reproducibility and statistical analyses of Fig. 3d. Independent western blot data using independent samples are shown. Statistical significance was calculated using two-way ANOVA followed by Tukey's multiple comparisons test (n = 3, biological replicates).

c, Reproducibility and statistical analyses of Fig. 3g. Independent western blot data using independent samples are shown. Statistical significance was calculated using two-way ANOVA followed by Tukey's multiple comparisons test (n = 3, biological replicates).

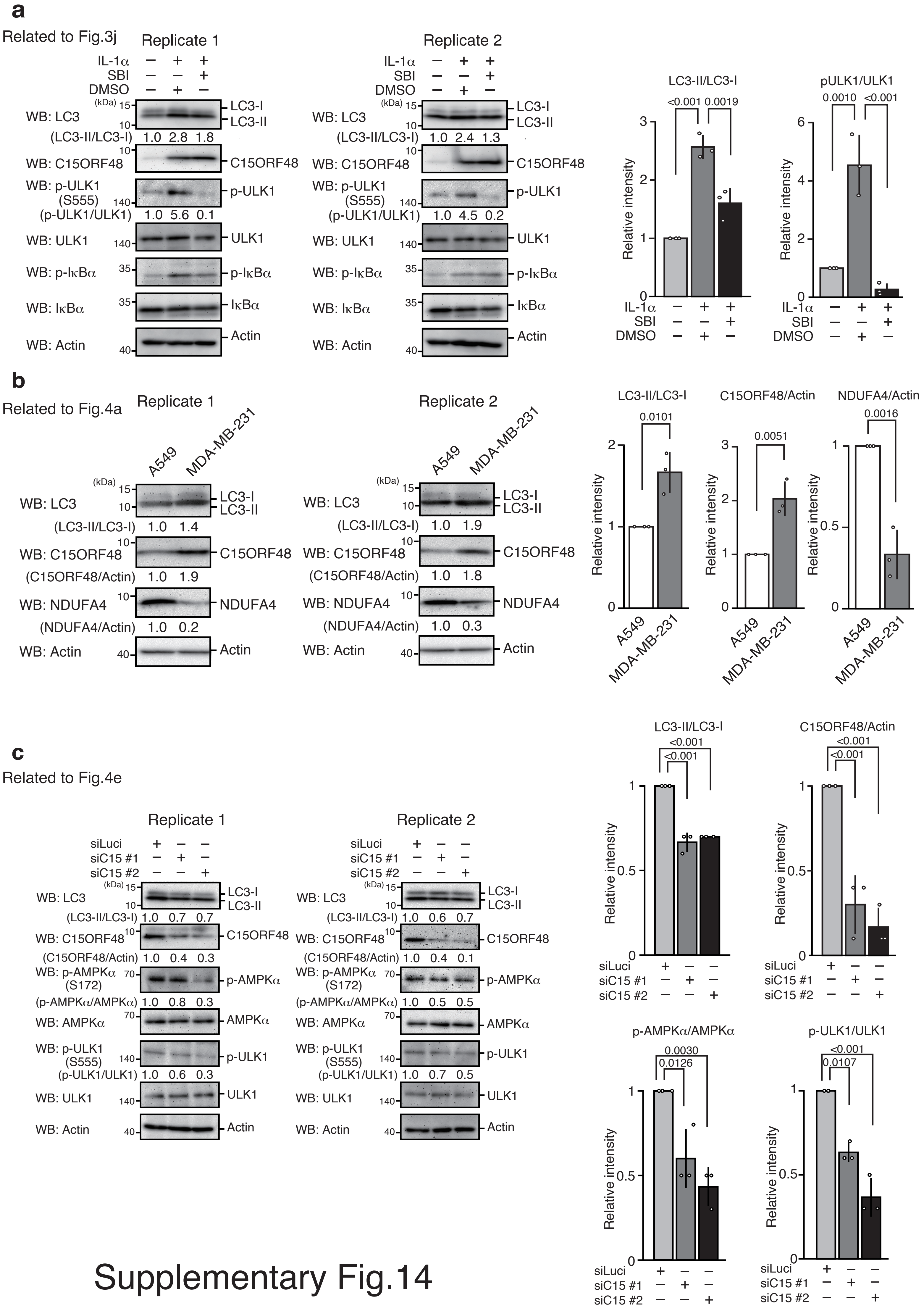

Supplementary Fig.14

Supplementary Fig. 14: Reproducibility and statistical analyses of western blotting.

a, Reproducibility and statistical analyses of Fig. 3j. Independent western blot data using independent samples are shown. Statistical significance was calculated using two-way ANOVA followed by Tukey's multiple comparisons test. N.S., Not Significant.

b, Reproducibility and statistical analyses of Fig. 4a. Independent western blot data using independent samples are shown. Statistical significance was calculated using two-tailed unpaired Student's *t*-test (n = 3, biological replicates).

c, Reproducibility and statistical analyses of Fig. 4e. Independent western blot data using independent samples are shown. Statistical significance was calculated using two-way ANOVA followed by Tukey's multiple comparisons test (n = 3, biological replicates).

a

Related to Fig.5c

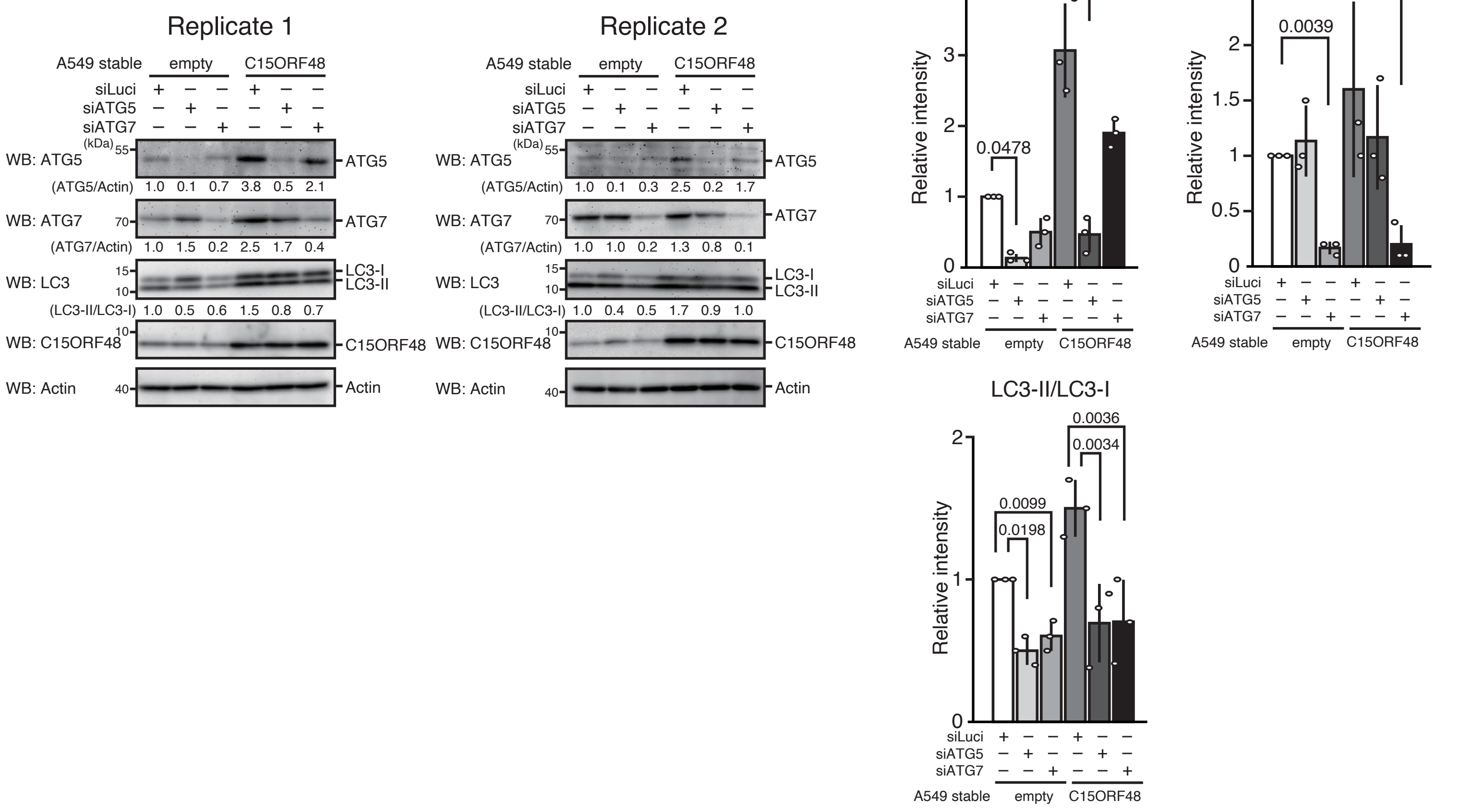

b

Related to Fig.5e

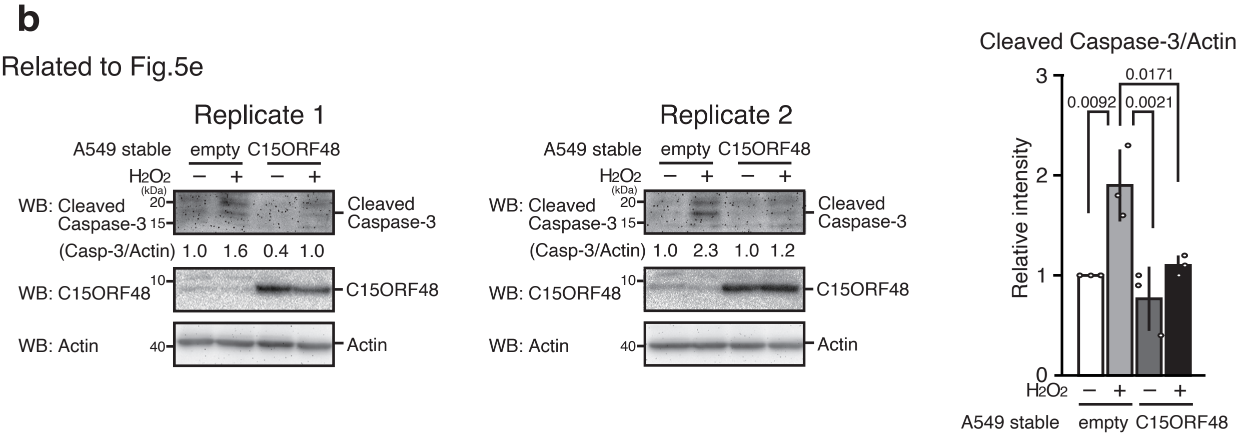

c

Related to Fig.5n

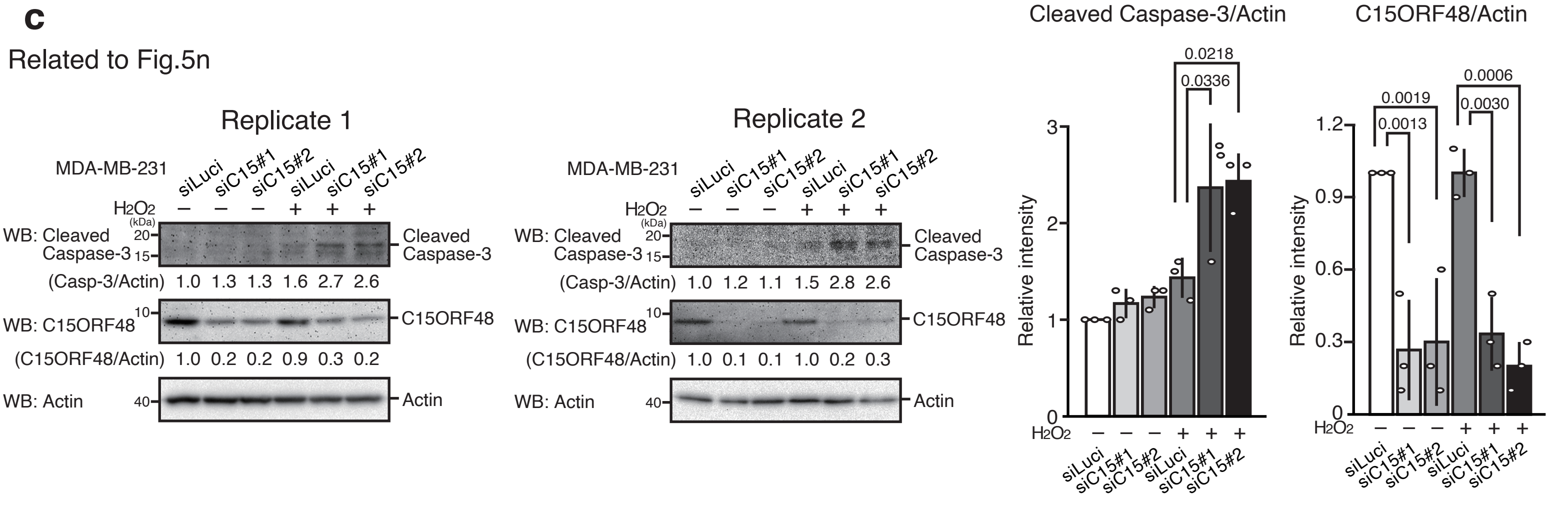

Supplementary Fig.15

Supplementary Fig. 15: Reproducibility and statistical analyses of western blotting.

a, Reproducibility and statistical analyses of Fig. 5c. Independent western blot data using independent samples are shown. Statistical significance was calculated using two-way ANOVA followed by Tukey's multiple comparisons test (n = 3, biological replicates). N.S., Not Significant.

b, Reproducibility and statistical analyses of Fig. 5e. Independent western blot data using independent samples are shown. Statistical significance was calculated using two-way ANOVA followed by Tukey's multiple comparisons test (n = 3, biological replicates).

c, Reproducibility and statistical analyses of Fig. 5n. Independent western blot data using independent samples are shown. Statistical significance was calculated using two-way ANOVA followed by Tukey's multiple comparisons test (n = 3, biological replicates).

a

Related to Suppl. Fig.1b

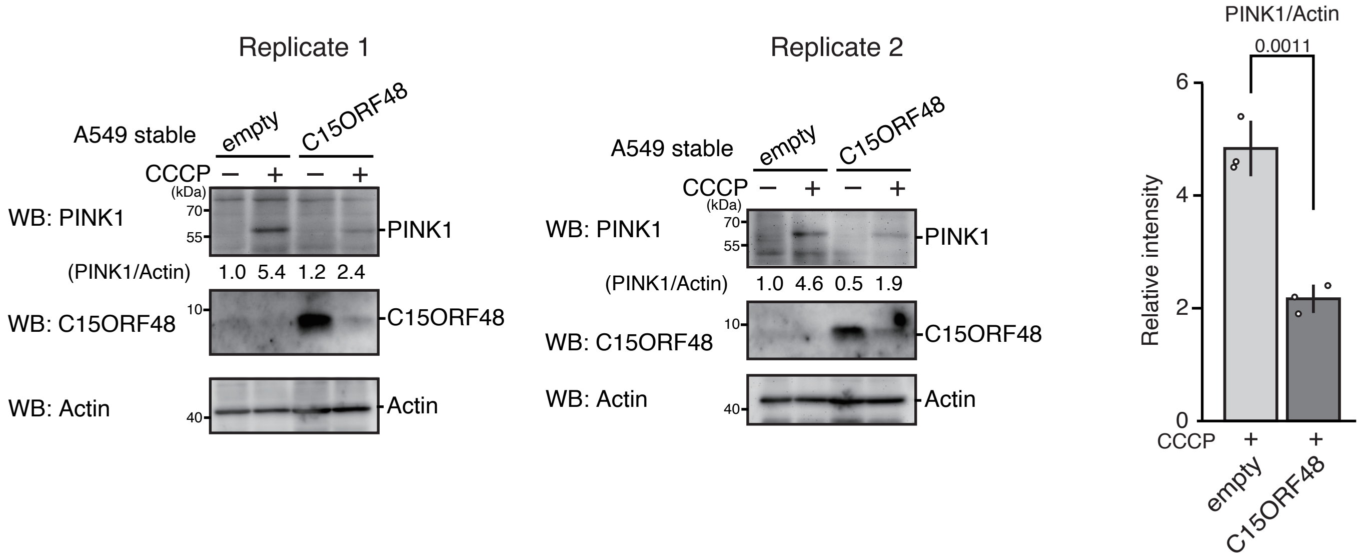

b

Related to Suppl. Fig.2b

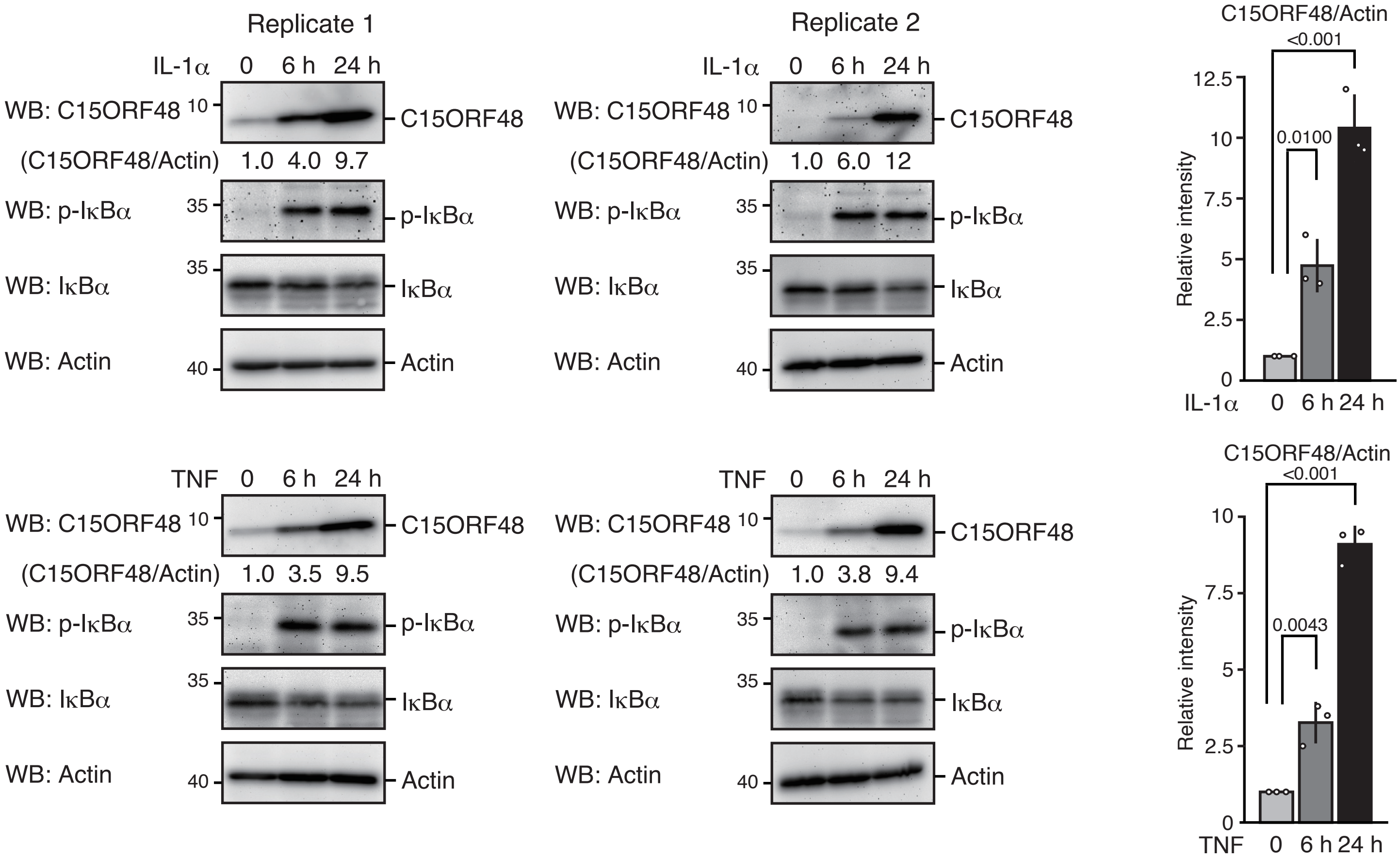

Supplementary Fig.16

Supplementary Fig. 16: Reproducibility and statistical analyses of western blotting.

a, Reproducibility and statistical analyses of Supplementary Fig. 1b. Independent western blot data using independent samples are shown. Statistical significance was calculated using two-tailed unpaired Student's *t*-test (n = 3, biological replicates). N.S., Not Significant.

b, Reproducibility and statistical analyses of Supplementary Fig. 2b. Independent western blot data using independent samples are shown. Statistical significance was calculated using two-way ANOVA followed by Tukey's multiple comparisons test (n = 3, biological replicates).

a

Related to Suppl. Fig.2d

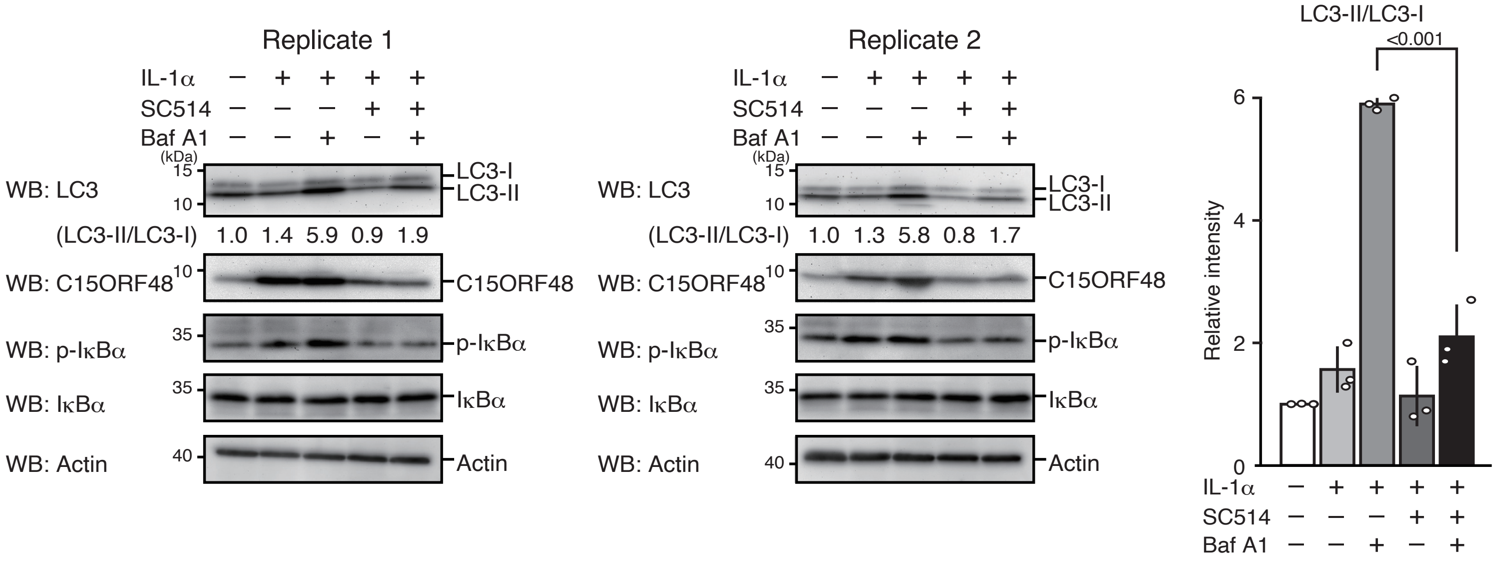

b

Related to Suppl. Fig.3a

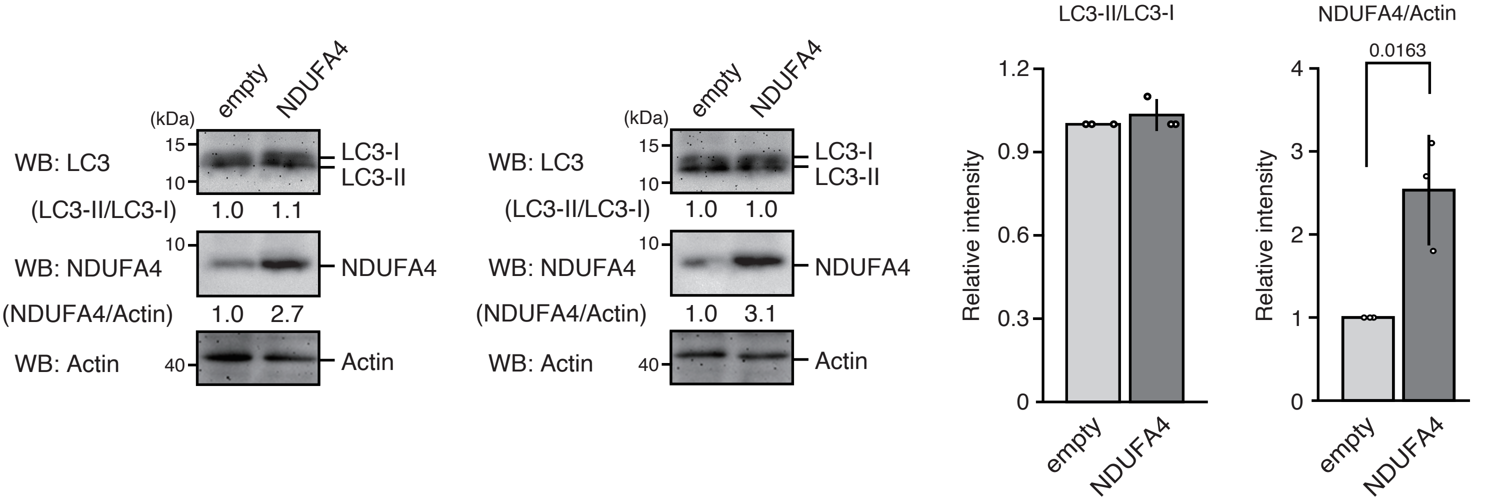

c

Related to Suppl. Fig.3b

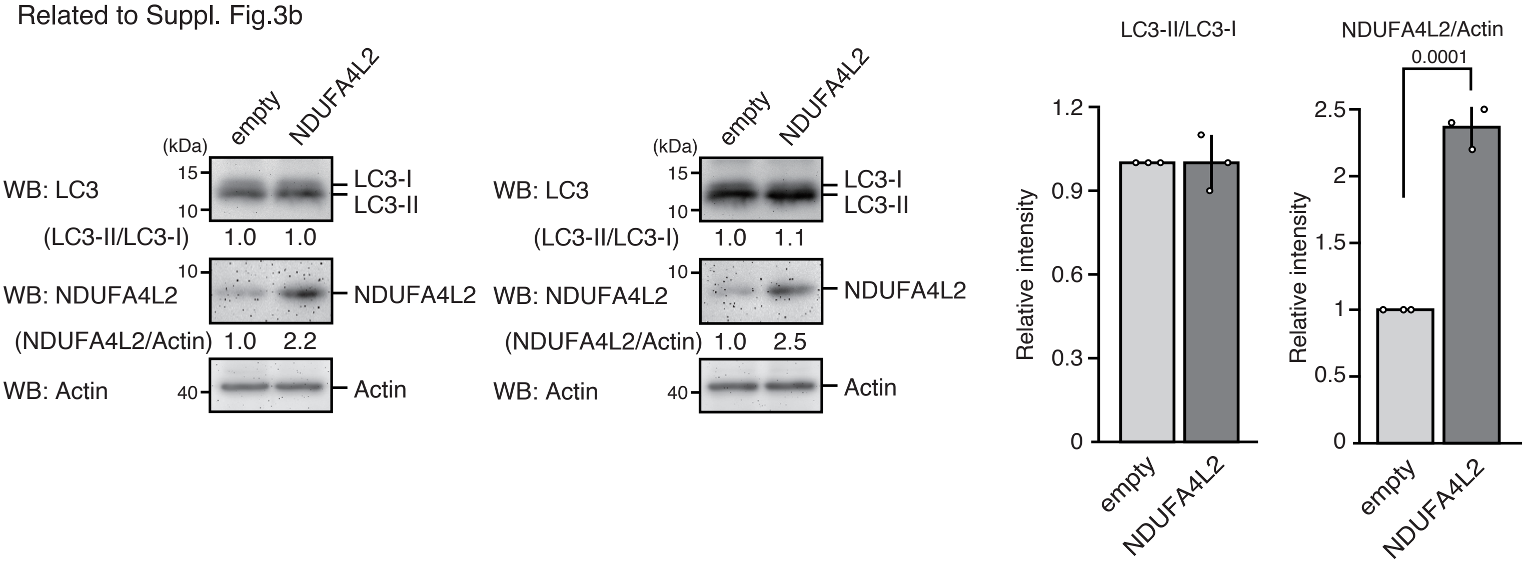

Supplementary Fig.17

Supplementary Fig. 17: Reproducibility and statistical analyses of western blotting.

a, Reproducibility and statistical analyses of Supplementary Fig. 2d. Independent western blot data using independent samples are shown. Statistical significance was calculated using two-way ANOVA followed by Tukey's multiple comparisons test (n = 3, biological replicates).

b, Reproducibility and statistical analyses of Supplementary Fig. 3a. Independent western blot data using independent samples are shown. Statistical significance was calculated using two-tailed unpaired Student's *t*-test (n = 3, biological replicates).

c, Reproducibility and statistical analyses of Supplementary Fig. 3b. Independent western blot data using independent samples are shown. Statistical significance was calculated using two-tailed unpaired Student's *t*-test (n = 3, biological replicates).

a

Related to Suppl. Fig.4d

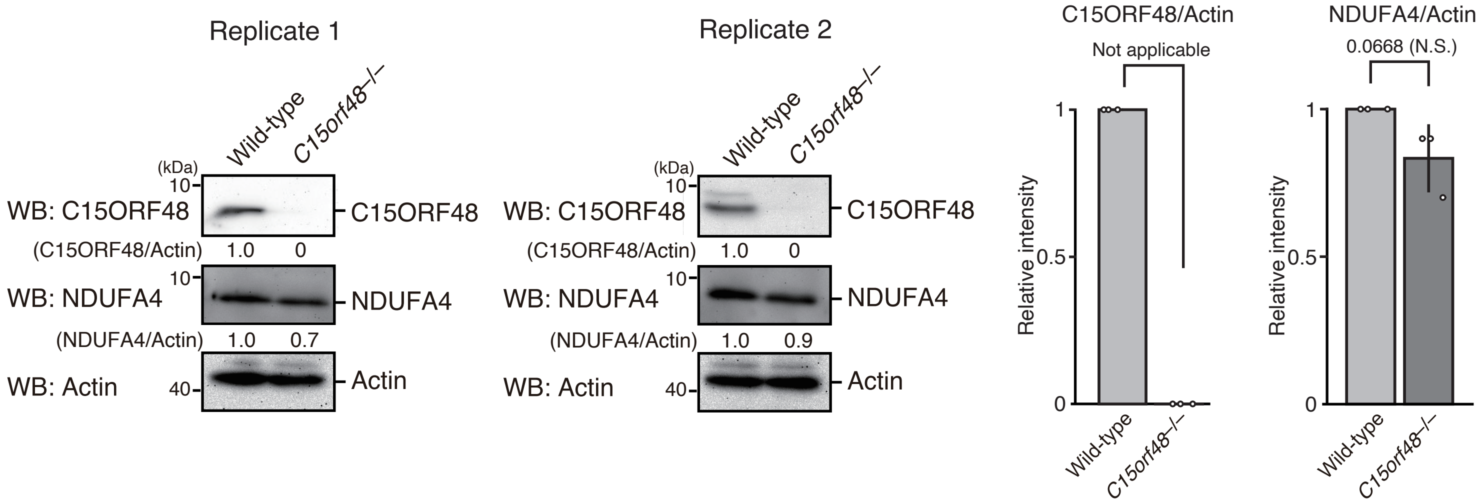

b

Related to Suppl. Fig.5a

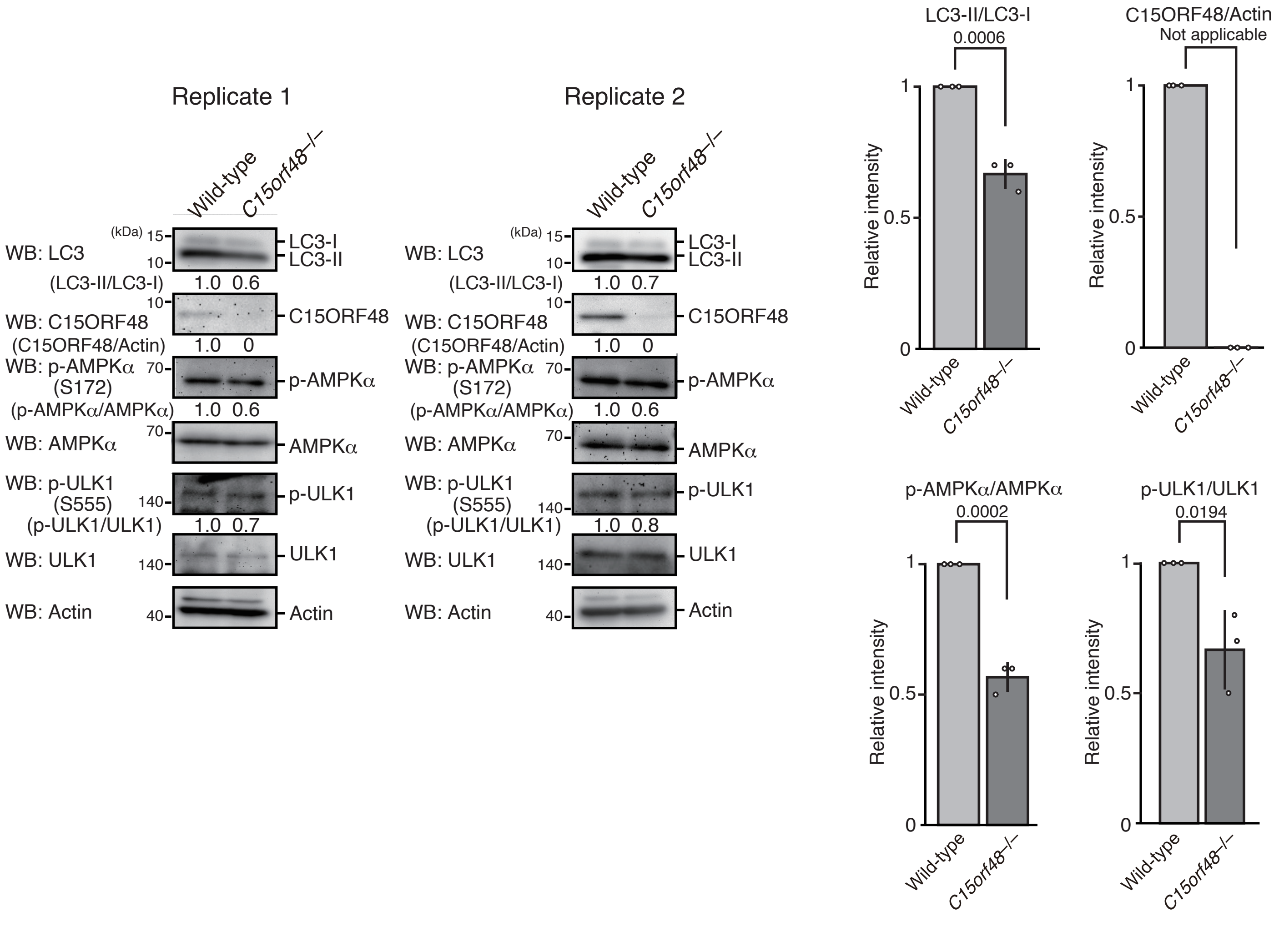

Supplementary Fig.18

Supplementary Fig. 18: Reproducibility and statistical analyses of western blotting.

a, Reproducibility and statistical analyses of Supplementary Fig. 4d. Independent western blot data using independent samples are shown. Statistical significance was calculated using two-way ANOVA followed by Tukey's multiple comparisons test (n = 3, biological replicates). N.S., Not Significant.

b, Reproducibility and statistical analyses of Supplementary Fig. 5a. Independent western blot data using independent samples are shown. Statistical significance was calculated using two-tailed unpaired Student's *t*-test (n = 3, biological replicates).

a

Related to Fig.1b, 3b, Suppl. Fig. 3c, 5b

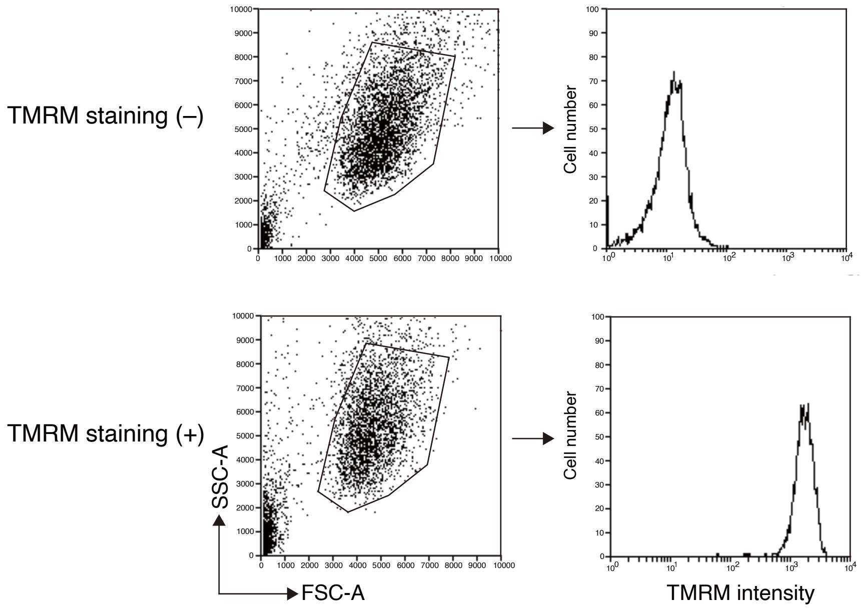

b

Related to Fig. 2h, Suppl. Fig. 1a

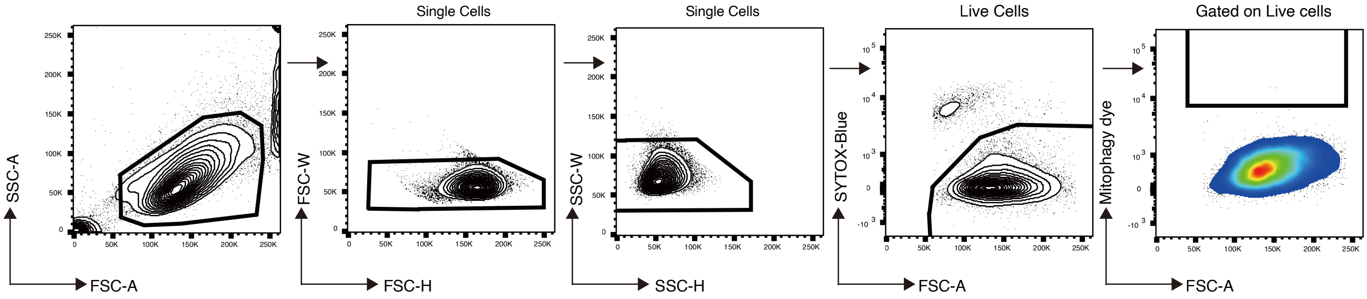

Supplementary Fig.19

Supplementary Fig. 19: Gating strategies of flow cytometry.

a, Gating strategy for analysis of TMRM intensity in Fig. 1b, 3b , Suppl. Fig. 3c, and 5d. In this sample gating, cells were gated in SSC-A and FSC-A dot plot to select viable cells, and then median fluorescent intensity of TMRM was determined in this population. SSC-A, side-scatter area; FSC-A, forward-scatter area.

b, Gating strategy of analysis of mitophagy dye-positive cells in Fig. 2h and Suppl. Fig. 1a. In this sample gating, cells were gated in SSC-A and FSC-A dot plot to viable cells and then gated in FSC-H/FSC-W and SSC-H/SSC-W dot plots to eliminate doublets. Dead cells were further eliminated by SYTOX-blue-staining, and then mitophagy dye-positive cells in viable cells were determined. FSC-H, forward-scatter height; FSC-W, forward-scatter width; SSC-H, side-scatter height; SSC-W, side-scatter width.

a

Related to Fig.6b, d

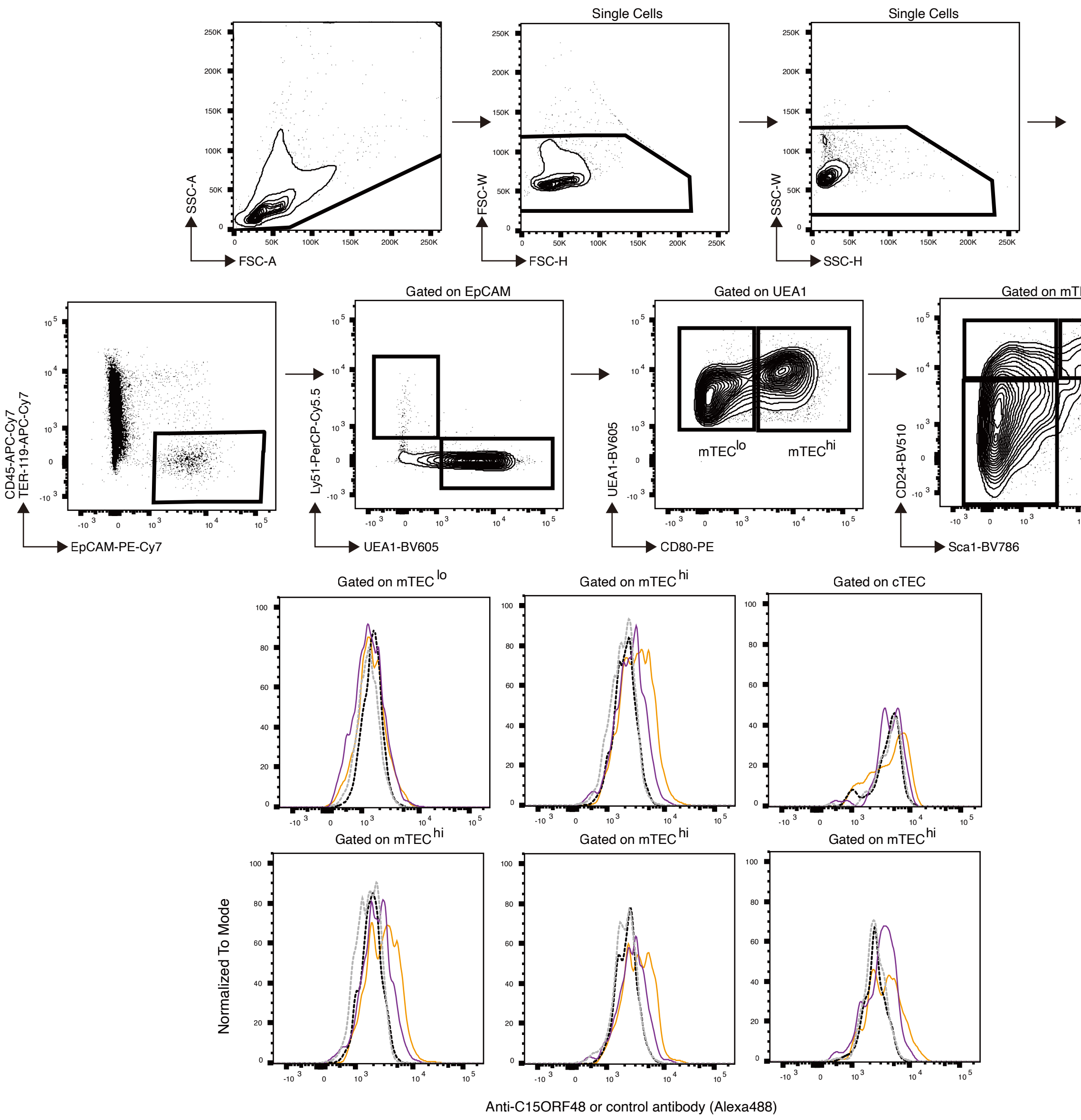

b

Related to Fig.6f

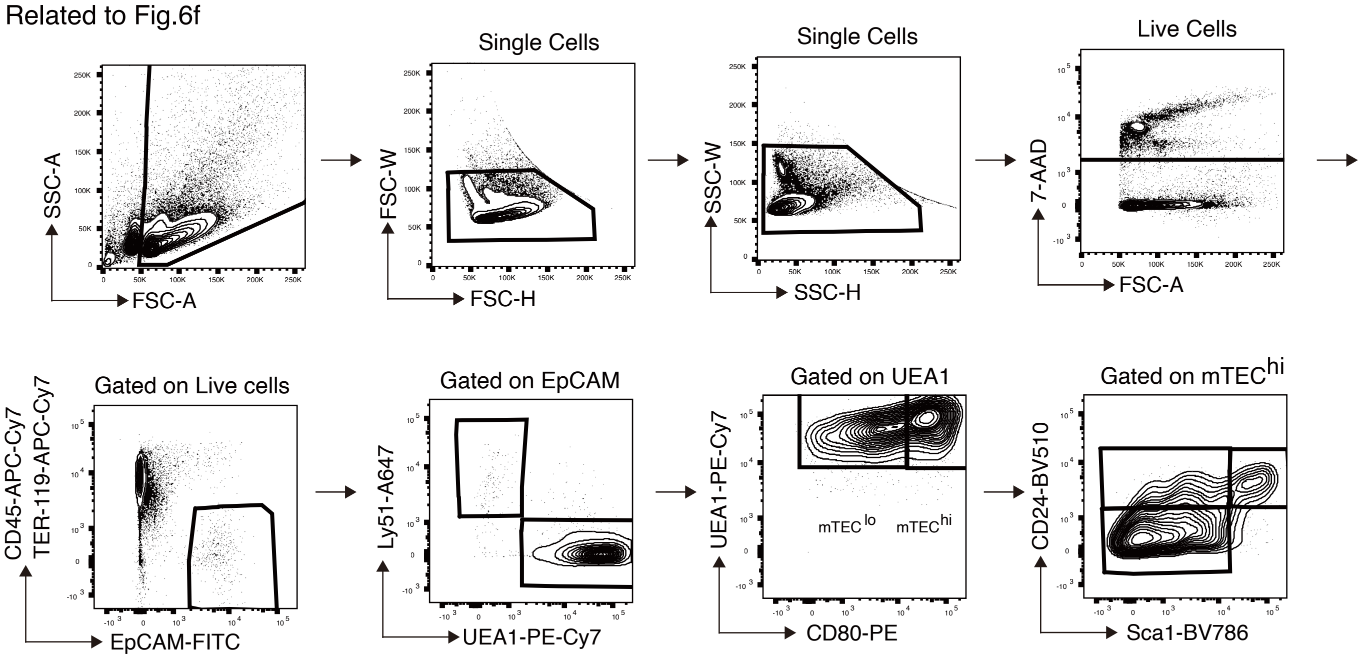

Supplementary Fig.20

Supplementary Fig. 20: Gating strategies of flow cytometry.

a, Gating strategy for analysis of cTECs and mTECs in Fig. 6b and 6d. In this sample gating, thymic cells were gated in SSC-A and FSC-A dot plot to select viable cells and then gated in FSC-H/FSC-W and SSC-H/SSC-W dot plots to eliminate doublets. Epithelial cells were detected by gating on EpCAM-positive and CD45/TER-119-negative cells. Epithelial cells were further divided into cTECs (Ly51-positive) and cTECs (UEA1-positive). mTECs were subdivided into $mTEC^{lo}$ and $mTEC^{hi}$ depending on CD80 expression levels. $mTEC^{hi}$ were classified into Early-Aire, Late-Aire, and Post-Aire mTECs depending on Sca1 and CD24 expression levels.

b, Gating strategy of analysis of cTECs and mTECs in Fig. 6f. In this sample gating, cells were gated in SSC-A and FSC-A dot plot to viable cells and then gated in FSC-H/FSC-W and SSC-H/SSC-W dot plots to eliminate doublets. Dead cells were further eliminated by 7-AAD-staining. cTECs and mTECs in this population were detected as in Suppl. Fig. 20a.

a

Related to Fig. 6g, Suppl. Fig. 9

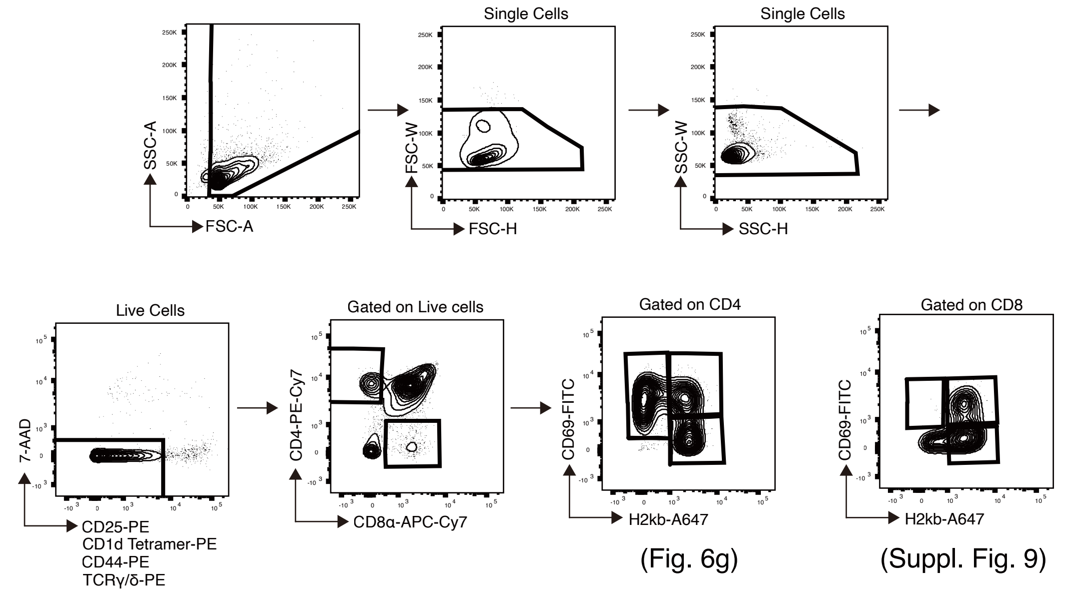

b

Related to Suppl. Fig. 7b

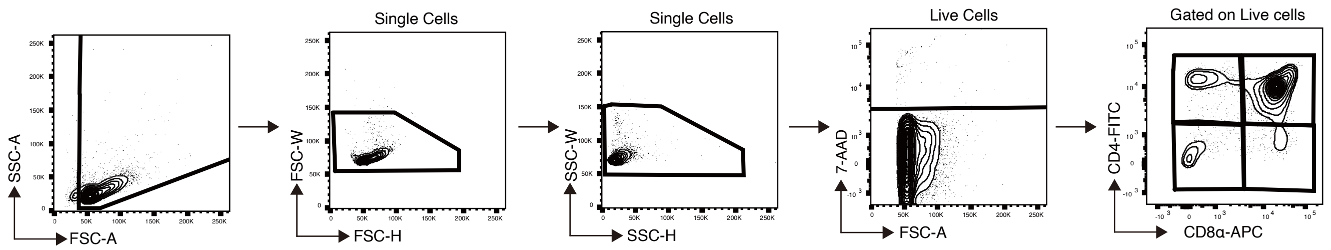

c

Related to Suppl. Fig. 8a

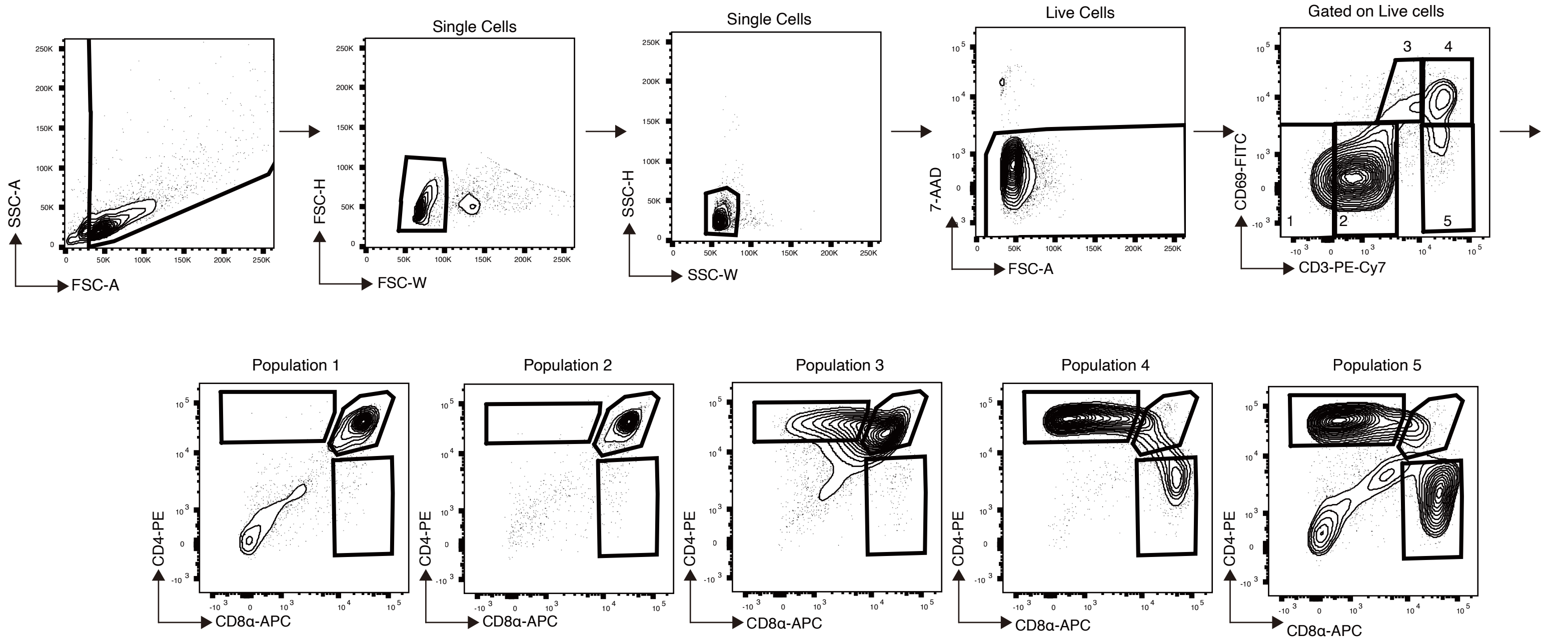

Supplementary Fig.21

Supplementary Fig. 21: Gating strategies of flow cytometry.

a, Gating strategy for analysis of SM, M1, and M2 T cells in Fig. 6g (CD4SP) and Suppl. Fig. 9 (CD8SP). In this sample gating, thymic cells were gated in SSC-A and FSC-A dot plot to select viable cells and then gated in FSC-H/FSC-W and SSC-H/SSC-W dot plots to eliminate doublets. Dead cells, Tregs, NKT cells, activated T cells, γ/δ T cells were removed by detecting 7-AAD, CD25, CD1d, CD44, and TCRγ/δ staining, respectively. CD4SP or SD8SP cells were detected in this gated population and then subdivided into SM, M1, and M2 cells depending on MHC class I (H2kb) and CD69 expression levels.

b, Gating strategy for analysis of CD4 and CD8 T cells in Suppl. Fig. 7b. In this sample gating, thymic cells were gated in SSC-A and FSC-A dot plot to select viable cells and then gated in FSC-H/FSC-W and SSC-H/SSC-W dot plots to eliminate doublets. Dead cells were further removed by detecting 7-AAD staining. DN, CD4SP, SD8SP, and DP cells were detected in this gated population by examining CD4 and CD8 expression levels.

c, Gating strategy for analysis of positive selection of thymocytes in Suppl. Fig. 8a. In this sample gating, thymic cells were gated in SSC-A and FSC-A dot plot to select viable cells and then gated in FSC-H/FSC-W and SSC-H/SSC-W dot plots to eliminate doublets. Dead cells were further removed by detecting 7-AAD staining. Subpopulations (population 1-5) in positive selection of thymocytes were detected by examining CD3 and CD69 expression levels in this gated population. CD4 and CD8 expression levels were further detected in each subpopulation.

a

Related to Suppl. Fig. 8b

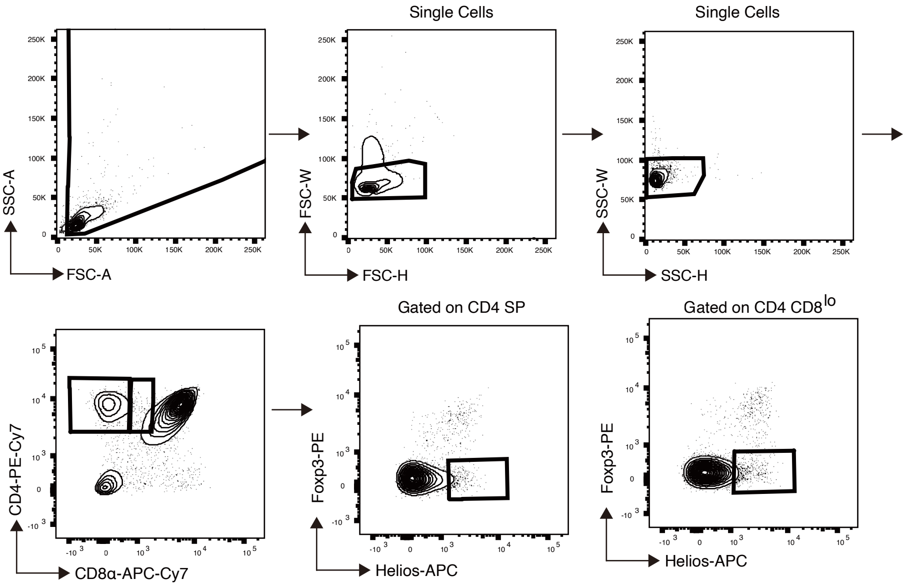

b

Related to Suppl. Fig. 8c

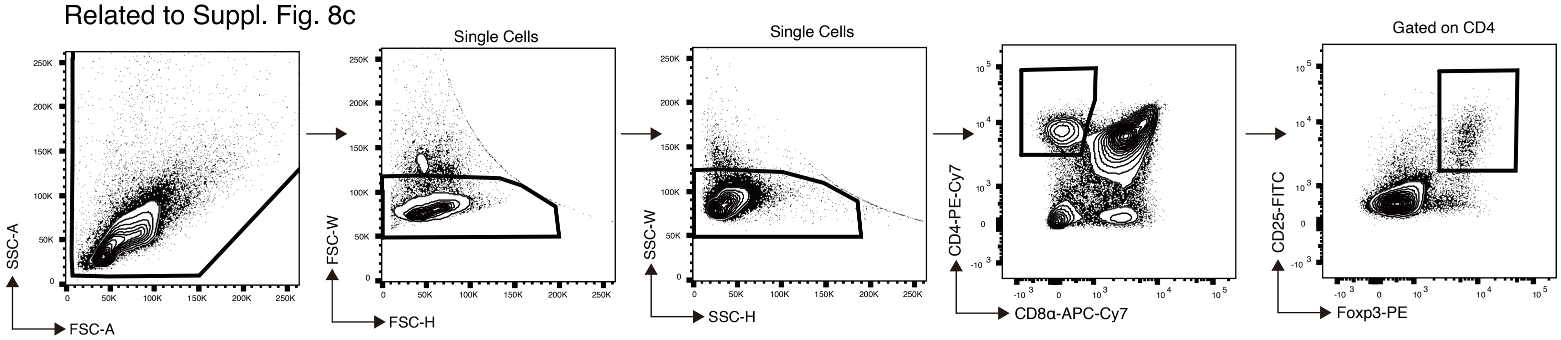

c

Related to Suppl. Fig. 10c, d

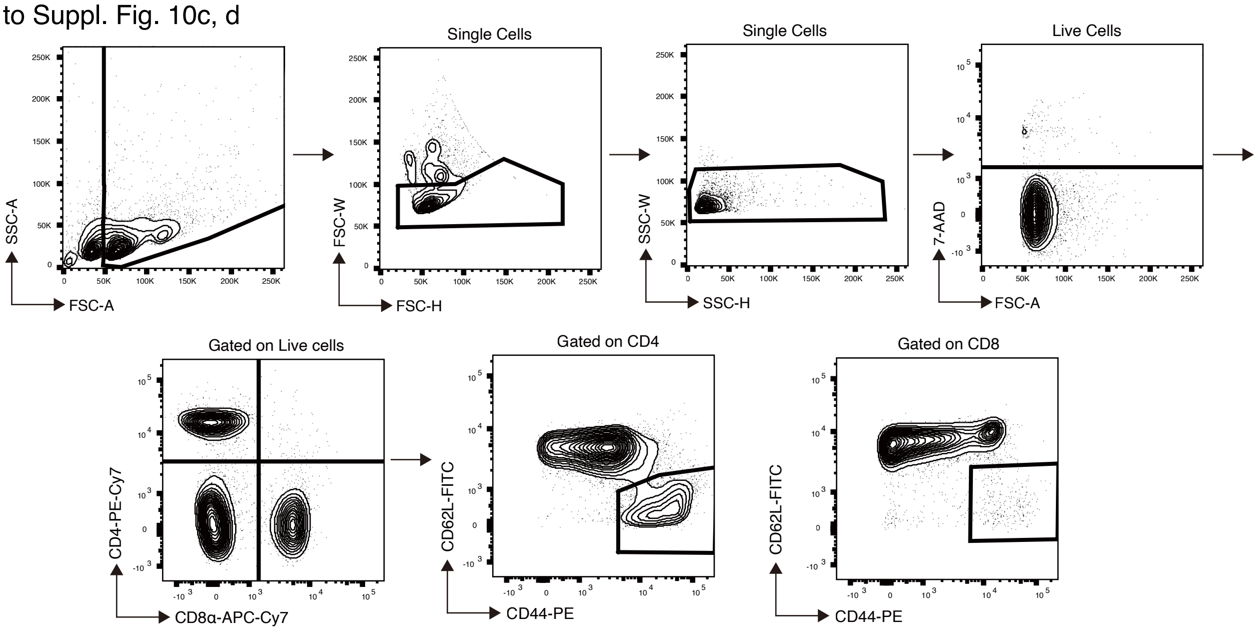

d

Related to Suppl. Fig. 10e

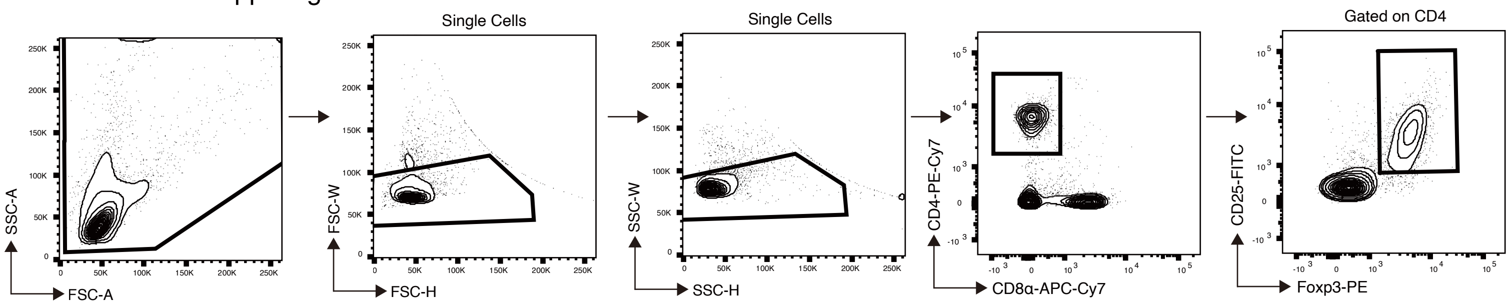

Supplementary Fig.22

Supplementary Fig. 22: Gating strategies of flow cytometry.

a, Gating strategy for analysis of negative selection of thymocytes in Suppl. Fig. 8b. In this sample gating, fixed thymic cells were gated in SSC-A and FSC-A dot plot and then gated in FSC-H/FSC-W and SSC-H/SSC-W dot plots to eliminate doublets. CD4SP cells were detected in this gated population, and Helios-positive and Foxp3-negative cells in CD4SP cells were detected as negative selection of thymocytes.

b, Gating strategy for analysis of Tregs in Suppl. Fig. 8c. In this sample gating, fixed thymic cells were gated in SSC-A and FSC-A dot plot and then gated in FSC-H/FSC-W and SSC-H/SSC-W dot plots to eliminate doublets. CD4SP cells were detected in this gated population, and Foxp3-positive and CD25-positive cells in CD4SP cells were detected as Tregs.

c, Gating strategy for analysis of memory T cells in Suppl. Fig. 10c and 10d. In this sample gating, cells in lymph nodes were gated in SSC-A and FSC-A dot plot to select viable cells and then gated in FSC-H/FSC-W and SSC-H/SSC-W dot plots to eliminate doublets. Dead cells were further removed by detecting 7-AAD staining. CD4SP and CD8SP cells were detected in this gated population, and CD44-positive and CD62L-negative cells in each population were detected as memory T cells.

d, Gating strategy for analysis of Tregs in Suppl. Fig. 10e. In this sample gating, fixed cells in lymph nodes were gated in SSC-A and FSC-A dot plot and then gated in FSC-H/FSC-W and SSC-H/SSC-W dot plots to eliminate doublets. CD4SP cells were detected in this gated population, and Foxp3-positive and CD25-positive cells in CD4SP cells were detected as Tregs.

Supplementary Table 1: Antibodies used in this study (Souce, Dilution, Catalog number)

Western blotting

NMES1 (C15ORF48) (Rabbit polyclonal) (Novus Biologicals, 1:1000, Cat#NBP1-98391)
NDUFA4 (Rabbit polyclonal) (EPIGENTEK, 1:1000, Cat#A73444)
Phospho-AMPKα (Thr172) (Rabbit monoclonal, 40H9) (Cell Signaling Technology, 1:1000, Cat#2535)
AMPKα (Rabbit monoclonal, D63G4) (Cell Signaling Technology, 1:1000, Cat#5832)
Phospho-ULK1 (Ser555) (Rabbit monoclonal, D1H4) (Cell Signaling Technology, 1:1000, Cat#5869)
ULK1 (Rabbit monoclonal, D8H5) (Cell Signaling Technology, 1:1000, Cat#8054)
Phospho-IκBα (Ser32) (Rabbit monoclonal, 14D4) (Cell Signaling Technology, 1:1000, Cat#2859)
IκBα (Mouse monoclonal, L35A5) (Cell Signaling Technology, 1:1000, Cat#4814)
LC3 (Mouse monoclonal, 8E10) (MBL, 1:1000, Cat#M186-3)
ATG5 (Mouse monoclonal, 4D3) (MBL, 1:1000, Cat#M153-3)
ATG7 (Rabbit polyclonal) (MBL, 1:1000, Cat#PM039)
Actin (Mouse monoclonal, C4) (Merck Millipore, 1:1000, Cat#MAB1501)
mTOR (Rabbit monoclonal, 7C10) (Cell Signaling Technology, 1:1000, Cat#2983)
Phospho-mTOR (Ser2481) (Rabbit polyclonal) (Cell Signaling Technology, 1:1000, Cat#2974)
Cleaved Caspase-3 (Asp175) (Rabbit polyclonal) (Cell Signaling Technology, 1:1000, Cat#9661)
NDUFA4L2 (Rabbit polyclonal) (ProteinTech, 1:1000, Cat#16480-1-AP)
PINK1 (Rabbit polyclonal) (Novus Biologicals, 1:1000, BC100-494)
Anti-Rabbit IgG, HRP-Linked Whole Ab Donkey (Cytiva, 1:1000, Cat#NA934)
Anti-Mouse IgG, HRP-Linked Whole Ab Sheep (Cytiva, 1:1000, Cat#NA931)

Immunocytochemistry

NMES1 (C15ORF48) (Rabbit polyclonal) (Novus Biologicals, 1:100, Cat#NBP1-98391)
LC3 (Rabbit polyclonal) (MBL, 1:300, Cat#PM036)
Alexa Fluor 488 goat anti-mouse IgG(H+L) (Thermo fisher Scientific, 1:300, Cat#A11029)
Alexa Fluor 546 goat anti-rabbit IgG(H+L) (Thermo fisher Scientific, 1:300, Cat#A11010)

Immunohistochemistry

GFP (Chicken polyclonal) (abcam, 1:300, Cat#ab13970)
Purified anti-Keratin 5 (Rabbit polyclonal) (Biolegend, 1:300, Cat#905504)
Purified anti-Keratin 8 (Rabbit polyclonal) (Developmental Stdies Hybridoma Bank, 1:200, Cat#TROMA-I)
Alexa Fluor 488 goat anti-chicken IgG(H+L) (Thermo fisher Scientific, 1:300, Cat#A11039)
Alexa Fluor 546 goat anti-rabbit IgG(H+L) (Thermo fisher Scientific, 1:300, Cat#A11010)

Flow cytometry

NMES1 (C15ORF48) (Rabbit polyclonal) (Novus Biologicals, 1:100, Cat#NBP1-98391)
Purified anti-mouse CD16/32 (Rat monoclonal, 2.4G2) (Biolegend, 1:200, Cat#101302)
APC/Cyanine7 anti-mouse CD45 (Rat monoclonal, 30-F11) (Biolegend, 1:200, Cat#103116)
FITC anti-mouse CD326 Ep-CAM (Rat monoclonal, G8.8) (Biolegend, 1:400, Cat#118208)
PE/Cyanine7 anti-mouse CD326 Ep-CAM (Rat monoclonal, G8.8) (Biolegend, 1:400, Cat#118215)
PerCP/Cyanine5.5 anti-mouse Ly51 (Rat monoclonal, 6C3) (Biolegend, 1:400, Cat#108315)
Alexa Fluor 647 anti-mouse Ly51 (Rat monoclonal, 6C3) (Biolegend, 1:400, Cat#108312)
PE anti-mouse CD80 (Armenian Hamster monoclonal, 16-10A1) (Biolegend, 1:400, Cat#104708)
Brilliant Violet 510 anti-mouse CD24 (Rat monoclonal, M1/69) (Biolegend, 1:300, Cat#101831)
Brilliant Violet 785 anti-mouse Ly-6A/E (Sca-I) (Rat monoclonal, D7) (Biolegend 1:300, Cat#108139)
PE/Cynine7 anti-mouse CD4 (Rat monoclonal, RM4-5) (Biolegend, 1:400, Cat#100528)
FITC Rat anti-mouse CD4 (Rat monoclonal, RM4-5) (BD Biosciences, 1:400, Cat#553047)
Alexa Fluor 647 anti-mouse CD8 (Rat monoclonal, 53-6.7) (Biolegend, 1:400, Cat#100724)
APC/Cyanine7 anti-mouse CD8 (Rat monoclonal, 53-6.7) (Biolegend, 1:400, Cat#100714)
FITC anti-mouse CD69 (Armenian Hamster monoclonal, H1.2F3) (Biolegend, 1:400, Cat#104506)
Alexa Fluor 647 anti-mouse H2-κb (Mouse monoclonal, AF6-88.5) (Biolegend, 1:400, Cat#116511)
PE anti-mouse/human CD44 (Rat monoclonal, IM7) (Biolegend, 1:400, Cat#103008)
PE Hamster anti-mouse γδT-Cell Receptor (Armenian Hamster monoclonal, GL3) (BD Biosciences, 1:200, Cat#553178)
FITC anti-mouse CD25 (Rat monoclonal, PC61) (Biolegend, 1:400, Cat#102006)
PE anti-mouse CD25 (Rat monoclonal, PC61) (Biolegend, 1:400, Cat#102007)
PE CD1d Tetramer (National Institute of Health provided, 1:50)
FITC anti-mouse CD62L (Rat monoclonal, MEL-14) (Biolegend, 1:400, Cat#104406)
APC anti-mouse CD357 (GITR) (Rat monoclonal, DTA-1) (Biolegend, 1:400, Cat#126312)
PE FOXP3 (Rat monoclonal, FJK-16s) (eBioscience, 1:400, Cat#12-5773-82)
APC anti-mouse/human Helios (Armenian Hamster monoclonal, 22F6) (Biolegend, 1:100, Cat#137221)
APC/Cyanine7 anti-mouse TER-119 (Rat monoclonal, TER119) (Biolegend, 1:200, Cat#116223)
Biotin anti-mouse CD3ε (Armenian Hamster monoclonal, 145-2C11) (Biolegend, 1:200, Cat#100304)
PE/Cyanine7 Streptavidin (Biolegend, 1:400, Cat#405206)
UEA1, biotinylated (Vector laboratories, 1:400, B-1065-2)
Isotype control (Rabbit IgG control) (R&D Systems, 1:200, AB-105-C)

Chromatin Immunoprecipitation (ChIP)

NF-κB RelA (Mouse monoclonal, F-6) (Santa Cruz Biotechnology, 4μL/sample, Cat#sc-8008)
Normal mouse IgG MOPC21 (Merck Millipore, 4μL/sample, Cat#M5284)
