# Supplementary material for: Mitochondrial protein C15ORF48 is a stress-independent inducer of autophagy that regulates oxidative stress and autoimmunity
Source: Nat Commun. 2024 Feb 1;15:953. doi: 10.1038/s41467-024-45206-1 (PMC10831050; doi:10.1038/s41467-024-45206-1)
Supplement: Supplementary file 3 — Reporting Summary [file 41467_2024_45206_MOESM3_ESM.pdf]

nature portfolio

Corresponding author(s): Taishin Akiyama, Noritaka Yamaguchi

Last updated by author(s): Dec 21, 2023

Reporting Summary

Nature Portfolio wishes to improve the reproducibility of the work that we publish. This form provides structure for consistency and transparency in reporting. For further information on Nature Portfolio policies, see our Editorial Policies and the Editorial Policy Checklist.

Statistics

For all statistical analyses, confirm that the following items are present in the figure legend, table legend, main text, or Methods section.

n/a	Confirmed	
☐	☒	The exact sample size (*n*) for each experimental group/condition, given as a discrete number and unit of measurement
☐	☒	A statement on whether measurements were taken from distinct samples or whether the same sample was measured repeatedly
☐	☒	The statistical test(s) used AND whether they are one- or two-sided<br>*Only common tests should be described solely by name; describe more complex techniques in the Methods section.*
☒	☐	A description of all covariates tested
☐	☒	A description of any assumptions or corrections, such as tests of normality and adjustment for multiple comparisons
☐	☒	A full description of the statistical parameters including central tendency (e.g. means) or other basic estimates (e.g. regression coefficient) AND variation (e.g. standard deviation) or associated estimates of uncertainty (e.g. confidence intervals)
☐	☒	For null hypothesis testing, the test statistic (e.g. *F*, *t*, *r*) with confidence intervals, effect sizes, degrees of freedom and *P* value noted<br>*Give P values as exact values whenever suitable.*
☒	☐	For Bayesian analysis, information on the choice of priors and Markov chain Monte Carlo settings
☒	☐	For hierarchical and complex designs, identification of the appropriate level for tests and full reporting of outcomes
☒	☐	Estimates of effect sizes (e.g. Cohen's *d*, Pearson's *r*), indicating how they were calculated

Our web collection on statistics for biologists contains articles on many of the points above.

Software and code

Policy information about availability of computer code

Data collection	Aria flow cytometer (BD Biosciences) for characterisation of cells<br>ChemiDoc XRS+ (Bio-rad) for chemical luminescent western blotting<br>Eco™ Real-PCR system (Illumina) and CFX96 Touch Real-Time PCR Detection System (BIO-rad) for qPCR<br>LSM780 (Zeiss) and SP8 (Leica) for confocal imaging<br>BZ-X710 (Keyence) for immunohistochemistry<br>Filter Max F5 microplate reader (Molecular Devices) for MTT, ATP, and glutathione assays
Data analysis	Data analysis and statistical analysis were performed using R version 4.2.0 and R Studio version 2022.02.3+492<br>Image J software (NIH, 1.53k) for image analysis<br>Quantity One (Bio-rad) for the quantification of band intensities of western blotting<br>FACS data was analyzed by FlowJo (BD Biosciences, v10)<br>Seurat v4.1.1 package (Satija Lab) for scRNA-seq plots

For manuscripts utilizing custom algorithms or software that are central to the research but not yet described in published literature, software must be made available to editors and reviewers. We strongly encourage code deposition in a community repository (e.g. GitHub). See the Nature Portfolio guidelines for submitting code & software for further information.

Data

Policy information about availability of data

All manuscripts must include a data availability statement. This statement should provide the following information, where applicable:

- Accession codes, unique identifiers, or web links for publicly available datasets
- A description of any restrictions on data availability
- For clinical datasets or third party data, please ensure that the statement adheres to our policy

We declare that all the data supporting the findings of this study are available in the paper and its supplementary information files. The DDBJ database of mouse thymic epithelial cells (DRA009125) was used for the scRNA-seq analysis. Source data are provided with this paper.

Research involving human participants, their data, or biological material

Policy information about studies with human participants or human data. See also policy information about sex, gender (identity/presentation), and sexual orientation and race, ethnicity and racism.

Reporting on sex and gender	N/A
Reporting on race, ethnicity, or other socially relevant groupings	N/A
Population characteristics	N/A
Recruitment	N/A
Ethics oversight	N/A

Note that full information on the approval of the study protocol must also be provided in the manuscript.

Field-specific reporting

Please select the one below that is the best fit for your research. If you are not sure, read the appropriate sections before making your selection.

☒ Life sciences ☐ Behavioural & social sciences ☐ Ecological, evolutionary & environmental sciences

For a reference copy of the document with all sections, see nature.com/documents/nr-reporting-summary-flat.pdf

Life sciences study design

All studies must disclose on these points even when the disclosure is negative.

Sample size	No statistically calculation methods were used to predetermine sample size estimates. Sample size was determined based on the experimental results obtained from our preliminary experiments and published papers (doi: 10.1096/fj.202100679RR, doi: 10.7554/eLife.73998). For animal studies, we chose standard sample sizes reported in the previous literature of mouse studies (doi: 10.7554/eLife.73998). The sample numbers (n) were described in each figure legends.
Data exclusions	No data was excluded from the analysis.
Replication	All experiments were performed at least three times to ensure that similar results were obtained. All experimental replications were indicated in the figure legends and quantification and statistical analysis section.
Randomization	Almost all of available mutant and control mice were randomly used for experiments without any selection. Randomized selection of mice from a large mouse colony was not done due to a limited space of mouse facility and animal welfare. For in vitro experiments, we conducted more than three independent experiments with different samples and confirmed reproducibility. As the results are strictly quantitative, randomization was not relevant in this study.
Blinding	Quantitation of inflammatory cell infiltration (Fig. 8c, 9c) and IgG deposits (Fig. 8e) were done in a double-blinded manner. Different researchers, who had no information about mouse genotypes, were prepared samples and scored inflammatory cell infiltration and IgG deposits. There was no blinding for other experiments. In these experiments, data analysis was strictly quantitative and were confirmed by multiple researchers who did not perform experiments. Therefore, blinding was not relevant in this study.

Reporting for specific materials, systems and methods

We require information from authors about some types of materials, experimental systems and methods used in many studies. Here, indicate whether each material, system or method listed is relevant to your study. If you are not sure if a list item applies to your research, read the appropriate section before selecting a response.

Materials & experimental systems

n/a	Involved in the study
☐	☒ Antibodies
☐	☒ Eukaryotic cell lines
☒	☐ Palaeontology and archaeology
☐	☒ Animals and other organisms
☒	☐ Clinical data
☒	☐ Dual use research of concern
☒	☐ Plants

Methods

n/a	Involved in the study
☒	☐ ChIP-seq
☐	☒ Flow cytometry
☒	☐ MRI-based neuroimaging

Antibodies

Antibodies used

All used antibodies were described in Supplementary Table S1.

Western blotting
NMES1 (C15ORF48) (Rabbit polyclonal) (Novus Biologicals, 1:1000, Cat#NBP1-98391)
NDUFA4 (Rabbit polyclonal) (EPIGENTEK, 1:1000, Cat#A73444)
Phospho-AMPK (Thr172) (Rabbit monoclonal, 40H9) (Cell Signaling Technology, 1:1000, Cat#2535)
AMPK (Rabbit monoclonal, D63G4) (Cell Signaling Technology, 1:1000, Cat#5832)
Phospho-ULK1 (Ser555) (Rabbit monoclonal, D1H4) (Cell Signaling Technology, 1:1000, Cat#5869)
ULK1 (Rabbit monoclonal, D8H5) (Cell Signaling Technology, 1:1000, Cat#8054)
Phospho-IκBα (Ser32) (Rabbit monoclonal, 14D4) (Cell Signaling Technology, 1:1000, Cat#2859)
IκBα (Mouse monoclonal, L35A5) (Cell Signaling Technology, 1:1000, Cat#4814)
LC3 (Mouse monoclonal, 8E10) (MBL, 1:1000, Cat#M186-3)
ATG5 (Mouse monoclonal, 4D3) (MBL, 1:1000, Cat#M153-3)
ATG7 (Rabbit polyclonal) (MBL, 1:1000, Cat#PM039)
Actin (Mouse monoclonal, C4) (Merck Millipore, 1:1000, Cat#MAB1501)
mTOR (Rabbit monoclonal, 7C10) (Cell Signaling Technology, 1:1000, Cat#2983)
Phospho-mTOR (Ser2481) (Rabbit polyclonal) (Cell Signaling Technology, 1:1000, Cat#2974)
Cleaved Caspase-3 (Asp175) (Rabbit polyclonal) (Cell Signaling Technology, 1:1000, Cat#9661)
NDUFA4L2 (Rabbit polyclonal) (ProteinTech, 1:1000, Cat#16480-1-AP)
PINK1 (Rabbit polyclonal) (Novus Biologicals, 1:1000, BC100-494)
Anti-Rabbit IgG, HRP-Linked Whole Ab Donkey (Cytiva, 1:1000, Cat#NA934)
Anti-Mouse IgG, HRP-Linked Whole Ab Sheep (Cytiva, 1:1000, Cat#NA931)

Immunocytochemistry
NMES1 (C15ORF48) (Rabbit polyclonal) (Novus Biologicals, 1:100, Cat#NBP1-98391)
LC3 (Rabbit polyclonal) (MBL, 1:300, Cat#PM036)
Alexa Fluor 488 goat anti-mouse IgG(H+L) (Thermo fisher Scientific, 1:300, Cat#A11029)
Alexa Fluor 546 goat anti-rabbit IgG(H+L) (Thermo fisher Scientific, 1:300, Cat#A11010)

Immunohistochemistry
GFP (Chicken polyclonal) (abcam, 1:300, Cat#ab13970)
Purified anti-Keratin 5 (Rabbit polyclonal) (Biolegend, 1:300, Cat#905504)
Purified anti-Keratin 8 (Rabbit polyclonal) (Developmental Stdies Hybridoma Bank, 1:200, Cat#TROMA-I)
Alexa Fluor 488 goat anti-chicken IgG(H+L) (Thermo fisher Scientific, 1:300, Cat#A11039)
Alexa Fluor 546 goat anti-rabbit IgG(H+L) (Thermo fisher Scientific, 1:300, Cat#A11010)

Flow cytometry
NMES1 (C15ORF48) (Rabbit polyclonal) (Novus Biologicals, 1:100, Cat#NBP1-98391)
Purified anti-mouse CD16/32 (Rat monoclonal, 2.4G2) (Biolegend, 1:200, Cat#101302)
APC/Cyanine7 anti-mouse CD45 (Rat monoclonal, 30-F11) (Biolegend, 1:200, Cat#103116)
FITC anti-mouse CD326 Ep-CAM (Rat monoclonal, G8.8) (Biolegend, 1:400, Cat#118208)
PE/Cyanine7 anti-mouse CD326 Ep-CAM (Rat monoclonal, G8.8) (Biolegend, 1:400, Cat#118215)
PerCP/Cyanine5.5 anti-mouse Ly51 (Rat monoclonal, 6C3) (Biolegend, 1:400, Cat#108315)
Alexa Fluor 647 anti-mouse Ly51 (Rat monoclonal, 6C3) (Biolegend, 1:400, Cat#108312)
PE anti-mouse CD80 (Armenian Hamster monoclonal, 16-10A1) (Biolegend, 1:400, Cat#104708)
Brilliant Violet 510 anti-mouse CD24 (Rat monoclonal, M1/69) (Biolegend, 1:300, Cat#101831)
Brilliant Violet 785 anti-mouse Ly-6A/E (Sca-l) (Rat monoclonal, D7) (Biolegend 1:300, Cat#108139)
PE/Cynine7 anti-mouse CD4 (Rat monoclonal, RM4-5) (Biolegend, 1:400, Cat#100528)
FITC Rat anti-mouse CD4 (Rat monoclonal, RM4-5) (BD Biosciences, 1:400, Cat#553047)
Alexa Fluor 647 anti-mouse CD8 (Rat monoclonal, 53-6.7) (Biolegend, 1:400, Cat#100724)
APC/Cyanine7 anti-mouse CD8 (Rat monoclonal, 53-6.7) (Biolegend, 1:400, Cat#100714)
FITC anti-mouse CD69 (Armenian Hamster monoclonal, H1.2F3) (Biolegend, 1:400, Cat#104506)
Alexa Fluor 647 anti-mouse H2-κb (Mouse monoclonal, AF6-88.5) (Biolegend, 1:400, Cat#116511)
PE anti-mouse/human CD44 (Rat monoclonal, IM7) (Biolegend, 1:400, Cat#103008)
PE Hamster anti-mouse γδT-Cell Receptor (Armenian Hamster monoclonal, GL3) (BD Biosciences, 1:200, Cat#553178)

FITC anti-mouse CD25 (Rat monoclonal, PC61) (Biolegend, 1:400, Cat#102006)
PE anti-mouse CD25 (Rat monoclonal, PC61) (Biolegend, 1:400, Cat#102007)
PE CD1d Tetramer (National Institute of Health provided, 1:50)
FITC anti-mouse CD62L (Rat monoclonal, MEL-14) (Biolegend, 1:400, Cat#104406)
APC anti-mouse CD357 (GITR) (Rat monoclonal, DTA-1) (Biolegend, 1:400, Cat#126312)
PE FOXP3 (Rat monoclonal, FJK-16s) (eBioscience, 1:400, Cat#12-5773-82)
APC anti-mouse/human Helios (Armenian Hamster monoclonal, 22F6) (Biolegend, 1:100, Cat#137221)
APC/Cyanine7 anti-mouse TER-119 (Rat monoclonal, TER119) (Biolegend, 1:200, Cat#116223)
Biotin anti-mouse CD3ε (Armenian Hamster monoclonal, 145-2C11) (Biolegend, 1:200, Cat#100304)
PE/Cyanine7 Streptavidin (Biolegend, 1:400, Cat#405206)
UEA1, biotinylated (Vector laboratories, 1:400, B-1065-2)
Isotype control (Rabbit IgG control) (R&D Systems, 1:200, AB-105-C)

Chromatin Immunoprecipitation (ChIP)
NF-κB RelA (Mouse monoclonal, F-6) (Santa Cruz Biotechnology, 4μL/sample, Cat#sc-8008)
Normal mouse IgG MOPC21 (Merch Millipore, 4μL/sample, Cat#M5284)

Validation

Western blotting
NMES1 (C15ORF48)
https://www.novusbio.com/products/nmes1-antibody_nbp1-98391
NDUFA4
https://www.epigentek.com/catalog/ndufa4-polyclonal-antibody-p-83750.html
Phospho-AMPKα (Thr172)
https://www.cellsignal.com/products/primary-antibodies/phospho-ampka-thr172-40h9-rabbit-mab/2535
AMPKα
https://www.cellsignal.com/products/primary-antibodies/ampka-d63g4-rabbit-mab/5832
Phospho-ULK1 (Ser555)
https://www.cellsignal.jp/products/primary-antibodies/phospho-ulk1-ser555-d1h4-rabbit-mab/5869
ULK1
https://www.cellsignal.jp/products/primary-antibodies/ulk1-d8h5-rabbit-mab/8054
Phospho-IκBα (Ser32)
https://www.cellsignal.jp/products/primary-antibodies/phospho-ikba-ser32-14d4-rabbit-mab/2859
IκBα
https://www.cellsignal.jp/products/primary-antibodies/ikba-l35a5-mouse-mab-amino-terminal-antigen/4814
LC3
https://ruo.mbl.co.jp/bio/dtl/A/index.html?pcd=M186-3
ATG5
https://ruo.mbl.co.jp/bio/dtl/A/index.html?pcd=M153-3
ATG7
https://ruo.mbl.co.jp/bio/dtl/A/index.html?pcd=PM039
Actin
https://www.sigmaaldrich.com/JP/ja/product/mm/mab1501?gclid=EAIaIQobChMIguyyp4-z_gIVx7CWCh36NA9kEAAYAiAAEgIaKPD_BwE
mTOR
https://www.cellsignal.jp/products/primary-antibodies/mtor-7c10-rabbit-mab/2983
Phospho-mTOR (Ser2481)
https://www.cellsignal.jp/products/primary-antibodies/phospho-mtor-ser2481-antibody/2974
Cleaved Caspase-3 (Asp175)
https://www.cellsignal.jp/products/primary-antibodies/cleaved-caspase-3-asp175-antibody/9661
NDUFA4L2
https://www.ptglab.co.jp/products/NDUFA4L2-Antibody-16480-1-AP.htm
PINK1
https://www.novusbio.com/products/pink1-antibody_bc100-494
Anti-Rabbit IgG, HRP-Linked Whole Ab Donkey
https://www.cytivalifesciences.co.jp/catalog/0428.html
Anti-Mouse IgG, HRP-Linked Whole Ab Sheep
https://www.cytivalifesciences.co.jp/catalog/0428.html

Immunocytochemistry
NMES1 (C15ORF48)
https://www.novusbio.com/products/nmes1-antibody_nbp1-98391
LC3 (Rabbit polyclonal)
https://www.mblintl.com/products/pm036/
Alexa Fluor 488 goat anti-mouse IgG(H+L)
https://www.thermofisher.com/antibody/product/Goat-anti-Mouse-IgG-H-L-Highly-Cross-Adsorbed-Secondary-Antibody-Polyclonal/A-11029
Alexa Fluor 546 goat anti-rabbit IgG(H+L)
https://www.thermofisher.com/antibody/product/Goat-anti-Rabbit-IgG-H-L-Cross-Adsorbed-Secondary-Antibody-Polyclonal/A-11010

Immunohistochemistry
GFP
https://www.abcam.co.jp/products/primary-antibodies/gfp-antibody-ab13970.html
Purified anti-Keratin 5
https://www.biolegend.com/ja-jp/search-results/purified-anti-keratin-5-antibody-13378
Purified anti-Keratin 8

https://dshb.biology.uiowa.edu/TROMA-I
Alexa Fluor 488 goat anti-mouse IgG(H+L)
https://www.thermofisher.com/antibody/product/Goat-anti-Mouse-IgG-H-L-Highly-Cross-Adsorbed-Secondary-Antibody-Polyclonal/A-11029
Alexa Fluor 546 goat anti-rabbit IgG(H+L)
https://www.thermofisher.com/antibody/product/Goat-anti-Rabbit-IgG-H-L-Cross-Adsorbed-Secondary-Antibody-Polyclonal/A-11010

Flow cytometry
NMES1 (C15ORF48)
https://www.novusbio.com/products/nmes1-antibody_nbp1-98391
Purified anti-mouse CD16/32
https://www.biolegend.com/ja-jp/products/purified-anti-mouse-cd16-32-antibody-190
APC/Cyanine7 anti-mouse CD45
https://www.biolegend.com/ja-jp/products/apc-cyanine7-anti-mouse-cd45-antibody-2530?GroupID=BLG1932
FITC anti-mouse CD326 Ep-CAM
https://www.biolegend.com/ja-jp/products/fitc-anti-mouse-cd326-ep-cam-antibody-4971?GroupID=BLG5748
PE/Cyanine7 anti-mouse CD326 Ep-CAM
https://www.biolegend.com/ja-jp/products/pe-cyanine7-anti-mouse-cd326-ep-cam-antibody-5303?GroupID=BLG6455
PerCP/Cyanine5.5 anti-mouse Ly51
https://www.biolegend.com/ja-jp/products/percp-cyanine5-5-anti-mouse-ly-51-antibody-13726
Alexa Fluor 647 anti-mouse Ly51
https://www.biolegend.com/ja-jp/products/alexa-fluor-647-anti-mouse-ly-51-antibody-3310?GroupID=BLG2523
PE anti-mouse CD80
https://www.biolegend.com/ja-jp/products/pe-anti-mouse-cd80-antibody-43
Brilliant Violet 510 anti-mouse CD24
https://www.biolegend.com/ja-jp/search-results/brilliant-violet-510-anti-mouse-cd24-antibody-9925
Brilliant Violet 785 anti-mouse Ly-6A/E (Sca-I)
https://www.biolegend.com/ja-jp/products/brilliant-violet-785-anti-mouse-ly-6a-e-sca-1-antibody-12077
PE/Cynine7 anti-mouse CD4
https://www.biolegend.com/ja-jp/products/pe-cyanine7-anti-mouse-cd4-antibody-1932?GroupID=BLG4211
FITC Rat anti-mouse CD4
https://www.bdbiosciences.com/en-nz/products/reagents/flow-cytometry-reagents/research-reagents/single-color-antibodies-ruo/fitc-rat-anti-mouse-cd4.553047
Alexa Fluor 647 anti-mouse CD8α
https://www.biolegend.com/ja-jp/products/alexa-fluor-647-anti-mouse-cd8a-antibody-2699
APC/Cyanine7 anti-mouse CD8α
https://www.biolegend.com/ja-jp/products/apc-cyanine7-anti-mouse-cd8a-antibody-2269
FITC anti-mouse CD69
https://www.biolegend.com/ja-jp/products/fitc-anti-mouse-cd69-antibody-264
Alexa Fluor 647 anti-mouse H2-kb
https://www.biolegend.com/ja-jp/products/alexa-fluor-647-anti-mouse-h-2kb-antibody-3238
PE anti-mouse/human CD44
https://www.biolegend.com/ja-jp/products/pe-anti-mouse-human-cd44-antibody-2206
PE Hamster anti-mouse γδ T-Cell Receptor
https://www.bdbiosciences.com/ja-jp/products/reagents/flow-cytometry-reagents/research-reagents/single-color-antibodies-ruo/pe-hamster-anti-mouse-t-cell-receptor.561997
FITC anti-mouse CD25
https://www.biolegend.com/ja-jp/products/fitc-anti-mouse-cd25-antibody-4511
PE anti-mouse CD25
https://www.biolegend.com/ja-jp/cell-separation/pe-anti-mouse-cd25-antibody-424
FITC anti-mouse CD62L (Rat monoclonal)
https://www.biolegend.com/ja-jp/products/fitc-anti-mouse-cd62l-antibody-384?GroupID=BLG10714
APC anti-mouse CD357 (GITR)
https://www.biolegend.com/ja-jp/products/apc-anti-mouse-cd357-gitr-antibody-4646?GroupID=BLG10765
PE FOXP3 (Rat monoclonal)
https://www.thermofisher.com/antibody/product/FOXP3-Antibody-clone-FJK-16s-Monoclonal/12-5773-82
APC anti-mouse/human Helios
https://www.biolegend.com/ja-jp/products/apc-anti-mouse-human-helios-antibody-6970
APC/Cyanine7 anti-mouse TER-119
https://www.biolegend.com/ja-jp/products/apc-cyanine7-anti-mouse-ter-119-erythroid-cells-antibody-3905
Biotin anti-mouse Cε
https://www.biolegend.com/ja-jp/products/biotin-anti-mouse-cd3epsilon-antibody-22
PE/Cyanine7 Streptavidin
https://www.biolegend.com/ja-jp/products/pe-cyanine7-streptavidin-1477
UEA1
https://vectorlabs.com/products/biotinylated-ulex-europaeus-agglutinin
Isotype control
https://www.rndsystems.com/products/normal-rabbit-igg-control_ab-105-c

Chromatin Immunoprecipitation (ChIP)
NF-κB RelA (Mouse monoclonal)
https://www.scbt.com/ja/p/nfkappab-p65-antibody-f-6
Normal mouse IgG MOPC21
https://www.sigmaaldrich.com/JP/ja/product/sigma/m5284

Eukaryotic cell lines

Policy information about cell lines and Sex and Gender in Research

Cell line source(s)	A549(Cat#CCL-185) and MDA-MB-231(Cat#HTB-26) cell lines were purchased from ATCC.
Authentication	Authentication was not performed for this study, because A549 and MDA-MB-231 were directly bought from ATCC.
Mycoplasma contamination	All cell lines used in this study were tested negative for mycoplasma.
Commonly misidentified lines (See ICLAC register)	No commonly misidentified cell lines were used.

Animals and other research organisms

Policy information about studies involving animals; ARRIVE guidelines recommended for reporting animal research, and Sex and Gender in Research

Laboratory animals	C57BL/6J mice (8–10 weeks old), BALB/c mice (8–10 weeks old), and BALB/c nude mice (6 weeks old) were purchased from Japan CLEA and used in this study. C15orf48 KO mice (C57BL/6J and BALB/c background) was generated in this study as described in the method section. GFP-LC3 mice (RBRC00806, C57BL/6 background) were obtained from RIKEN BRC. Rag1 KO mice (C57BL/6 background) were obtained from the Department of Animal Management at RIKEN. Mice were maintained under 12-hour light/12-hour dark cycle. The room temperature was regulated at 20°C and humidity was controlled at 50%.<br><br>For in vivo autophagy analysis, 4-week-old wild-type or C15orf48 KO mice crossed with GFP-LC3 mice were used (C57BL/6 background). For flow cytometric analysis, 4-week-old or 21-week-old wild-type or C15orf48 KO mice were used (C57BL/6 background). For autoantibody analysis, sera from 21-week-old wild-type or C15orf48 KO mice were reacted with tissue sections from Rag1 KO mice (C57BL/6 background). For infiltration and IgG deposits analyses, 21-week-old wild-type or C15orf48 KO mice were used (C57BL/6 background). For thymic transplantation experiments, fetal thymic cells (E15.5) from wild-type or C15orf48 KO mice were transplanted into renal capsules of 6-week-old nude mice (BALB/c background).
Wild animals	No wild animals were used in this study.
Reporting on sex	Only female mice were used for immunological experiments to exclude the effects of sex difference on immunological phenotypes.
Field-collected samples	No field-collected samples were used in this study.
Ethics oversight	All animal experiments were approved by the Institutional Animal Care and Use Committee of RIKEN Yokohama Branch (2018-075) and performed in accordance with Guidelines of the Institutional Animal Care and Use Committee at RIKEN.

Note that full information on the approval of the study protocol must also be provided in the manuscript.

Flow Cytometry

Plots

Confirm that:

☒ The axis labels state the marker and fluorochrome used (e.g. CD4-FITC).

☒ The axis scales are clearly visible. Include numbers along axes only for bottom left plot of group (a 'group' is an analysis of identical markers).

☒ All plots are contour plots with outliers or pseudocolor plots.

☒ A numerical value for number of cells or percentage (with statistics) is provided.

Methodology

Sample preparation	Thymic cells were collected by gently tweezing the organs with forceps in ice-cold PBS supplemented with 1% FBS.
Instrument	Aria flow cytometer
Software	FACS Flowjo v10 was used to analyze flow cytometry data.
Cell population abundance	Double positive thymocytes, single positive CD4, CD8 thymocytes, and double negative thymocytes; 97-99%<br>TER119- or CD45-negative and Epicam-positive population; 0.3-0.4%
Gating strategy	Doubles were excluded using FSC/SSC gates. Dead cells were excluded by gating on 7-AAD negative cells. Gating strategies are shown in the manuscript.

☒ Tick this box to confirm that a figure exemplifying the gating strategy is provided in the Supplementary Information.
